# Supplementary material for: Kauro, a graph-based chatbot for high-fidelity information transmission conversations
Source: medRxiv. 2026 Feb 2:2026.01.30.25342358. Preprint. [Version 1] doi: 10.64898/2026.01.30.25342358 (PMC12889763; doi:10.64898/2026.01.30.25342358)
Supplement: Supplement 2 [file media-2.pdf]

Full Consent Chat Transcript

Main

Node ID: CaLvKv4

Parents: start

Children: m84n5MM

Kauro: I’m Kauro, your automated Medical Information Assistant from the University of California, Irvine (UCI). I’m inviting you to participate in the **Pediatric Mendelian Genomics Research Center (PMGRC)** research study.

Kauro: I’m here to tell you all about the PMGRC study and help you decide if you want to participate

---

Node ID: m84n5MM

Parents: CaLvKv4

Children: de5ZDgm

User Transcript: 👋 Hi Kauro!

Node ID: de5ZDgm

Parents: m84n5MM

Children: PJwkbgi

Kauro: Some of the things I will review with you are: why we are doing this research, what you will be expected to do if you enroll, and possible risks and benefits

Kauro: This information will help you choose whether or not to participate

---

Node ID: PJwkbgi

Parents: de5ZDgm

Children: oS9m8Ua

User Transcript: Great!

Node ID: oS9m8Ua

Parents: PJwkbgi

Children: me5RCBw, iLpXdAS

Kauro: Our conversation usually takes about 30 minutes. At the end, if you choose to enroll, you can sign the consent.

Kauro: This link will expire two weeks after it was sent to you. You can always take a break and come back to this link, during the two weeks, to complete enrollment.

Node ID: me5RCBw

Parents: oS9m8Ua

Children: a6JKRdh

User Transcript: Okay

Node ID: a6JKRdh

Parents: iLpXdAS, me5RCBw

Children: WwRBQik

Kauro: All of the information we discuss will also be described in more detail in our consent document

Kauro: You can only sign the consent form electronically after reviewing it with me or with a study team member

Kauro: You can access an example of the consent document by clicking this link, but please remember to return here to complete our chat

---

Node ID: WwRBQik

Parents: a6JKRdh

Children: XcPGk57

User Transcript: Can I look at the consent document after we chat?

Node ID: XcPGk57

Parents: WwRBQik

Children: JPVkFCr

Kauro: Yes, you can come back here to access the link later, and I can also provide the link again at the end of the chat

---

Node ID: JPVkFCr

Parents: XcPGk57

Children: ZjTLunQ

User Transcript: Okay, great

Node ID: ZjTLunQ

Parents: JPVkFCr

Children: fWXv5QS

Kauro: Whether or not you choose to participate in the study, the team at UCI may review your interactions with me, Kauro, to better understand how people interact with me and how I can improve

---

Node ID: fWXv5QS

Parents: ZjTLunQ

Children: S2nH9zZ

User Transcript: Ok, sounds good

Node ID: S2nH9zZ

Parents: fWXv5QS

Children: 9DgwYTz, DdxFUae, BxD4wrq

Kauro: If you are ready, we can start going through the information together right now!

Kauro: If you would rather review the consent later or you would rather review it with a study team member, just let me know!

Node ID: 9DgwYTz

Parents: S2nH9zZ

Children: Q8yaxQc

User Transcript: I'd like to continue chatting with Kauro now

---

Node ID: Q8yaxQc

Parents: 9DgwYTz, 87q8gWG

Children: M4CaAt5

Kauro: Okay!

Kauro: Let's do this!

Kauro: Please be sure to take notes and write down any questions you have as we go through this chat. You will have the option to talk to a study team member at the end. ✏️

Kauro: If you have any questions that are not answered during this chat, we will put you in touch with a team member to discuss them further

---

Node ID: M4CaAt5

Parents: Q8yaxQc

Children: FADCStr

User Transcript: Sounds good! 👍

Node ID: FADCStr

Parents: M4CaAt5

Children: Vew9XnD, Bkyp7hD

Kauro: The goal of this study is to help identify the cause or causes of genetic health conditions

Kauro: You are being invited to join this study because you or someone in your family has a suspected genetic health condition (or a health issue that might be related to genes)

Kauro: We often enroll multiple family members (people related by blood) in the study

---

Node ID: Vew9XnD

Parents: FADCStr

Children: KMirb2i

User Transcript: Okay

Node ID: KMirb2i

Parents: RXBDyqd, Vew9XnD

Children: dM9iaFR

Kauro: Ideally, we would enroll **the person with the suspected genetic health condition** as well as **both of their biological parents** (mom and dad), if possible.

Kauro: So, if one or both parents are able to enroll, that would be great!

Kauro: You can still participate if family members are not available

---

Node ID: dM9iaFR

Parents: KMirb2i

Children: E3C8G5Y

User Transcript: Good to know!

Node ID: E3C8G5Y

Parents: dM9iaFR

Children: C8baGp6

Kauro: Each person who enrolls in the study will have to go through this study consent process

Kauro: You can enroll yourself and/or your children in this chat today. We can send a separate chat link to any other adults who want to enroll

---

Node ID: C8baGp6

Parents: E3C8G5Y

Children: CPf9CCz

User Transcript: Okay

Node ID: CPf9CCz

Parents: C8baGp6

Children: BBfTzRw, CAwKwCW

Kauro: For children who are 6 years old or younger, a parent or guardian can complete the consent on their behalf

Kauro: For children ages 7 to 17, (or for adults who are under a person's legal guardianship), a parent or guardian must complete the official consent

Kauro: We will also need to talk directly to the child (if they are capable) to get their agreement about participating. This is called assent

Kauro: People who are age 18 or older need to go through the consent process themselves unless they are under another person's legal guardianship

---

Node ID: BBfTzRw

Parents: CPf9CCz

Children: USfk36C

User Transcript: Okay

Node ID: USfk36C

Parents: CAwKwCW, BBfTzRw

Children: b5nYNf6

Kauro: Can you tell me who in your family might consider enrolling in this study? (check all that apply, including any parents or other adults who might enroll)

Node ID: Ey8gJrc

Parents: L5xPJtN

Children: KR89wri, QXNa2cz

Kauro: You're welcome 😀

Kauro: Now, let's take a moment to talk a little bit about genetics.

Kauro: Do you want to keep it simple or get a little more science-y?

Node ID: KR89wri

Parents: Ey8gJrc, WnXoffX

Children: hBC6sGH

User Transcript: Science-y please 🤓

Node ID: hBC6sGH

Parents: KR89wri

Children: EcgsbMN

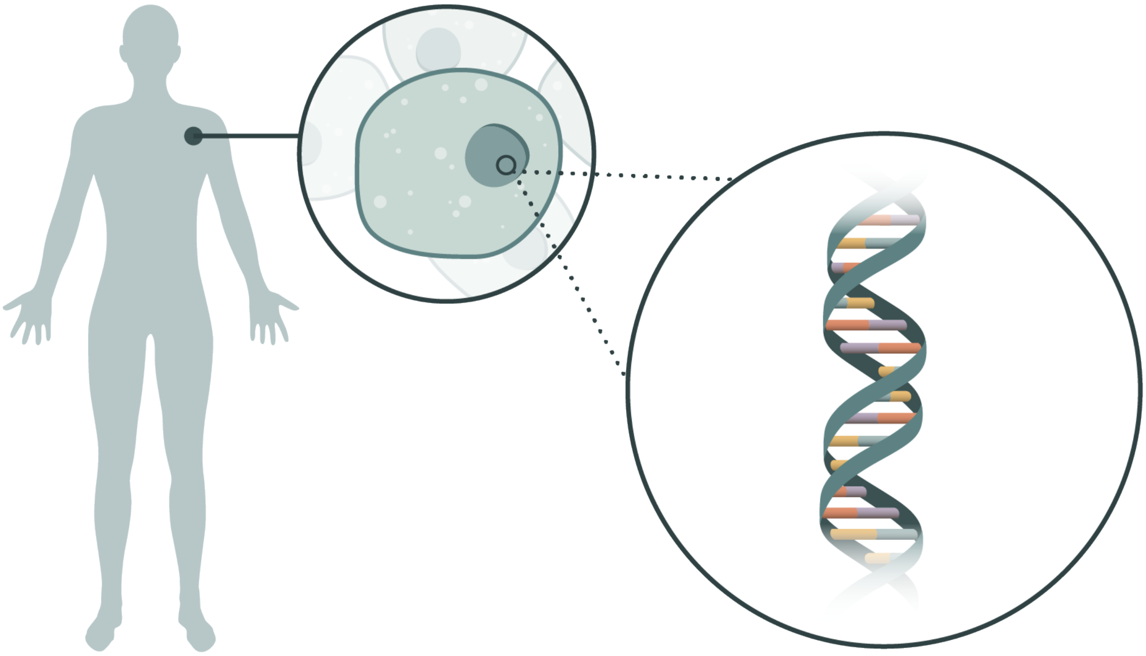

Kauro: I was hoping you would say that!

Kauro: You may already know that your body is made of trillions of cells. DNA provides the instructions to tell our bodies how to grow and develop

---

Node ID: EcgsbMN

Parents: hBC6sGH

Children: SfPWkep

User Transcript: Ah, okay

Node ID: SfPWkep

Parents: EcgsbMN

Children: AonpoZW

Kauro: Our DNA uses specific letters (A, T, C, and G) to write these instructions

Kauro: A little section of the code might look something like this:

ATGCGCTAGCTCCTAGCTAGCCTAGCTAGCTAACGCTAGCCTCGTAGACTACGATCGCTAAGCTAGC

Kauro: Our genetic code is actually over 3 billion letters long!

Node ID: AonpoZW

Parents: SfPWkep

Children: 45kz8CE

User Transcript: That’s complicated

Node ID: 45kz8CE

Parents: AonpoZW

Children: QjTtL5s, 9ZmfvBQ

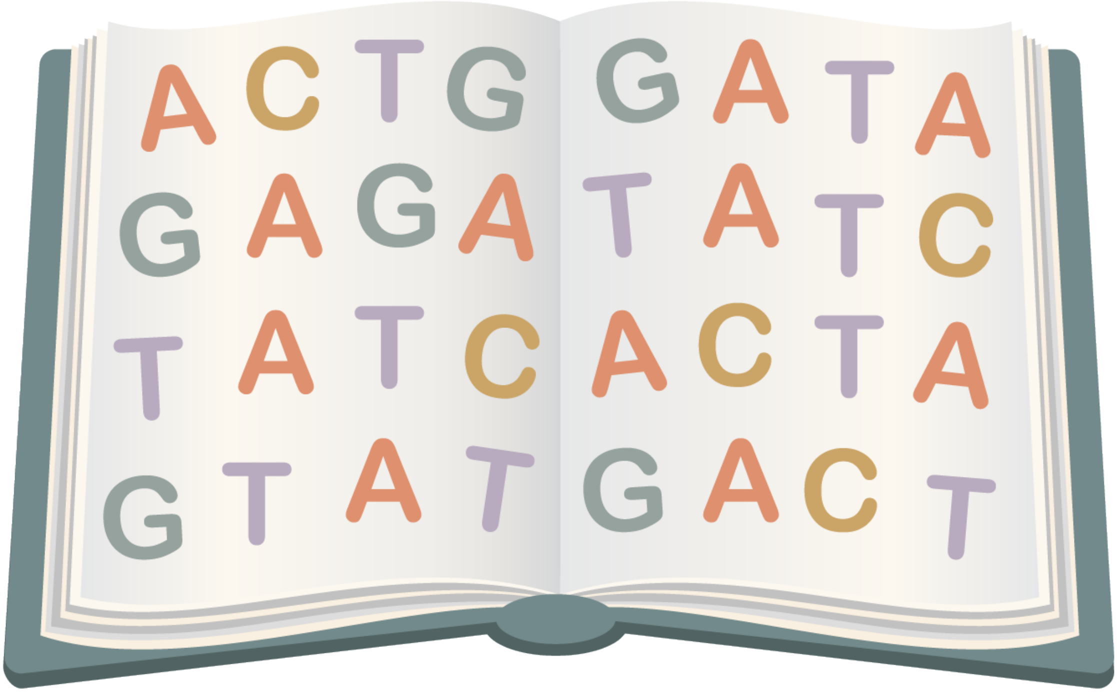

Kauro: I know this sounds complicated! Your cells know how the read these instructions. They use your genetic code like a cookbook

Kauro: Our DNA is organized into specific sections, as if they are individual recipes. These sections, or the recipes, are called genes

---

Node ID: QjTtL5s

Parents: 45kz8CE

Children: BF3k6W7

User Transcript: This sounds familiar

Node ID: BF3k6W7

Parents: 9ZmfvBQ, QjTtL5s

Children: 8Pvoowc

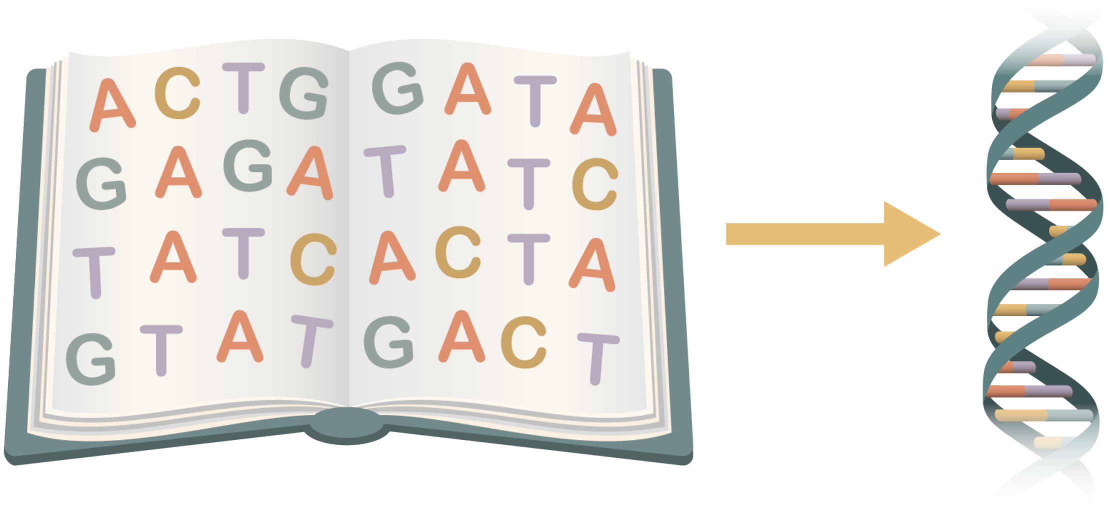

Kauro: In a cookbook, each recipe gives instructions for making a different dish

Kauro: In your cells, each gene provides the instructions for molecules called RNA, and each RNA makes a different protein

---

Node ID: 8Pvoowc

Parents: BF3k6W7

Children: WQFz456

User Transcript: That’s interesting

Node ID: WQFz456

Parents: 8Pvoowc

Children: kvTbHhw

Kauro: Your body uses proteins to do many important things

Kauro: For example, some proteins build our tissues, like muscles and nerves. Other proteins help our body break down food and make energy

---

Node ID: kvTbHhw

Parents: WQFz456

Children: PpUbXu4

User Transcript: That’s cool!

Node ID: PpUbXu4

Parents: kvTbHhw

Children: TfVxQFn

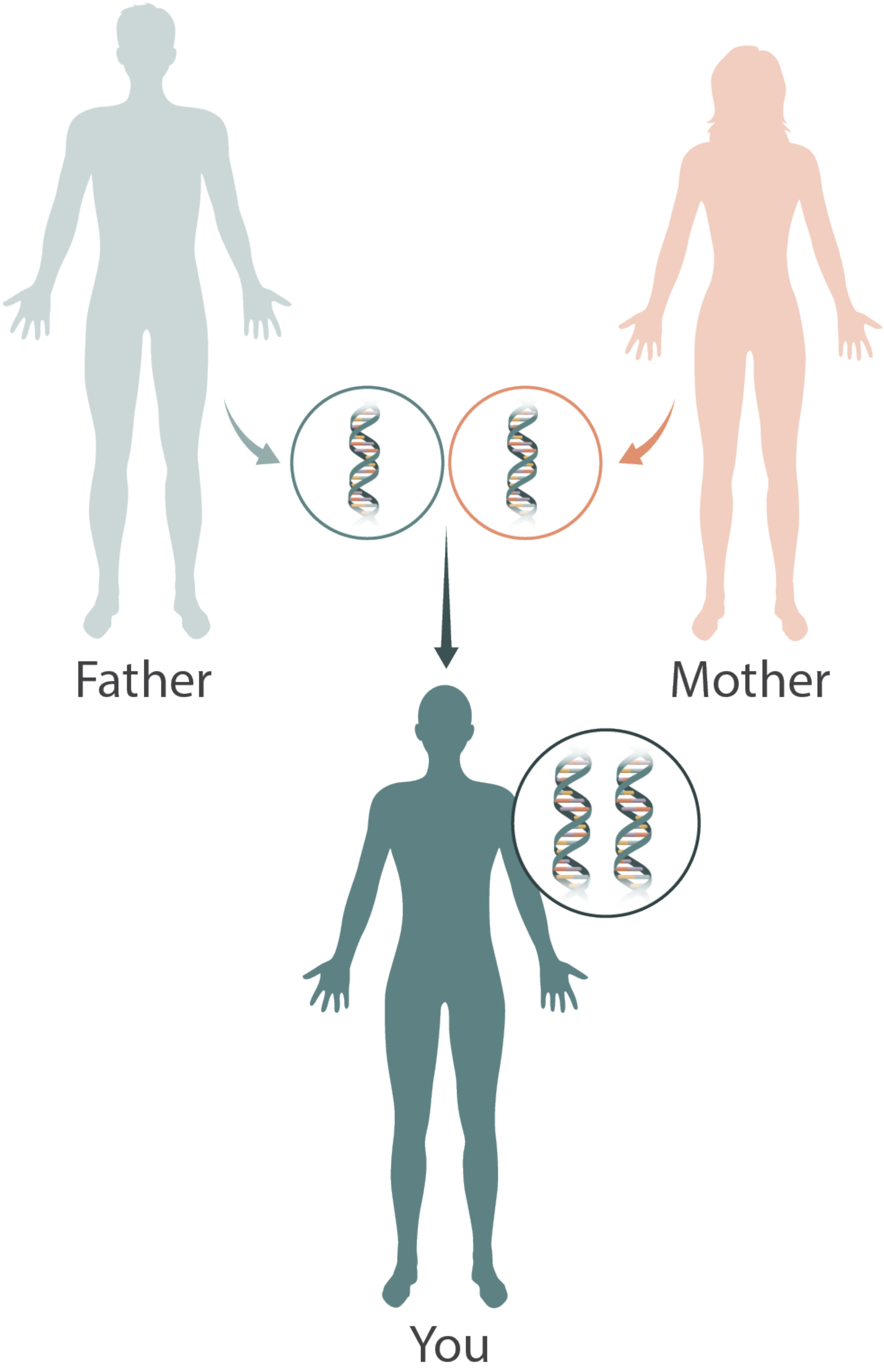

Kauro: I know, right?

Kauro: You typically have 2 copies of each gene - getting one from each of your parents. The 2 copies work together to make you who you are

---

Node ID: TfVxQFn

Parents: PpUbXu4

Children: HVeQxH2

User Transcript: Got it

Node ID: HVeQxH2

Parents: TfVxQFn

Children: Vnd4AC6

Kauro: People can have different versions of the same gene. This is what makes each person unique

Kauro: We call these different versions of a gene “variants”

Node ID: Vnd4AC6

Parents: HVeQxH2

Children: c85Yw2k

User Transcript: What is a variant?

Node ID: c85Yw2k

Parents: Vnd4AC6

Children: iumYvk4

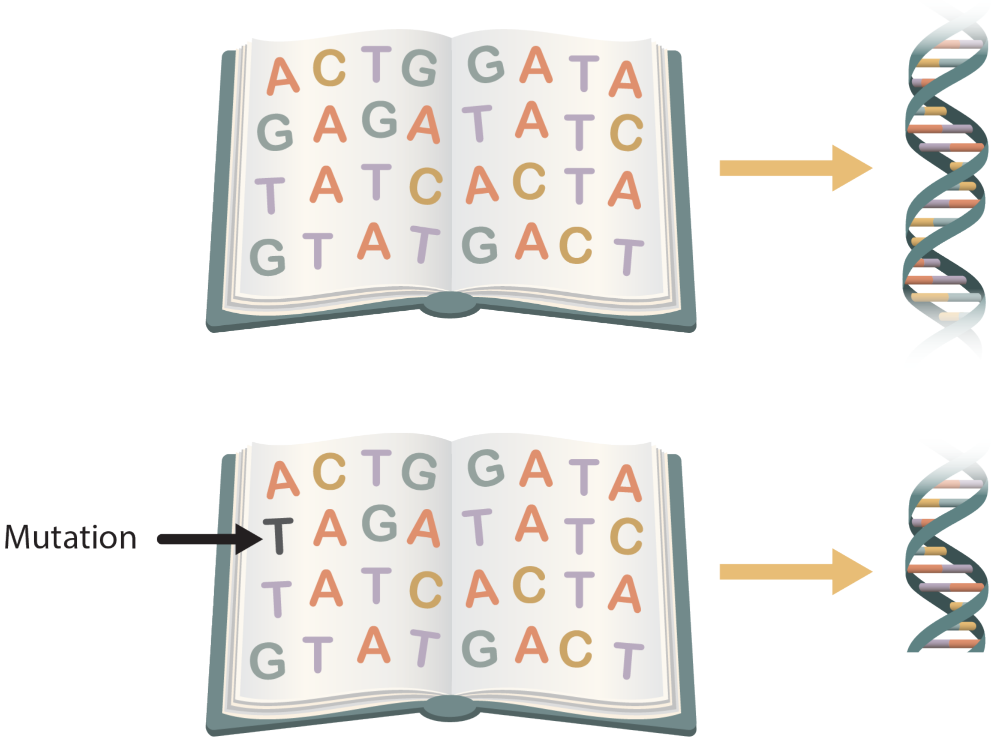

Kauro: Remember how we said your genes are like recipes?

Kauro: When genes have misspellings or changes in them, we call that a variant. This is like changing an ingredient in a recipe. The end product may turn out differently than you expect or may not work at all

---

Node ID: iumYvk4

Parents: c85Yw2k

Children: 7i6JiiZ

User Transcript: I see

Node ID: 7i6JiiZ

Parents: iumYvk4

Children: ZcynmTH

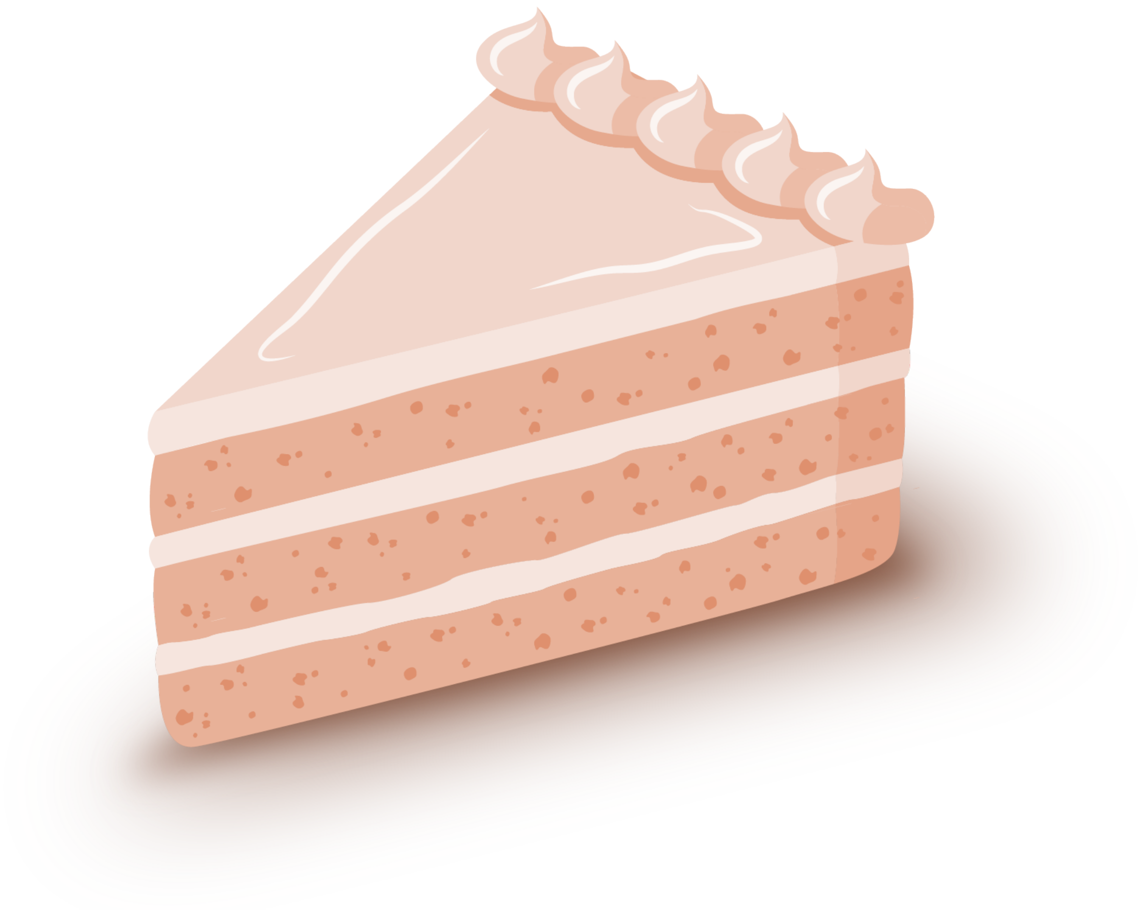

Kauro: Imagine that you are baking a cake and the recipe tells you to use salt instead of sugar, or strawberries instead of chocolate!

Kauro: That's going to affect the cake!

Node ID: ZcynmTH

Parents: 7i6JiiZ

Children: EE6kxBa

User Transcript: Got it

Node ID: EE6kxBa

Parents: Ti3ZavP, ZcynmTH

Children: iMcrZ9h, iVCEBuL

Kauro: Genetic variants are what make each person unique

Kauro: Some variants may change a person's eye color, height, or hair color, while others may change a person's development, heart function, or kidney function

---

Node ID: iMcrZ9h

Parents: EE6kxBa

Children: 8Z6qtgu

User Transcript: Interesting

Node ID: 8Z6qtgu

Parents: iVCEBuL, iMcrZ9h

Children: Zgg7vB9

Kauro: When someone has a health issue or symptoms caused by a genetic change (or “variant”), this can cause a genetic disorder

Kauro: When we suspect that a patient has a genetic disorder, we often do genetic testing to try and find the change, or variant, that is causing the symptoms

Kauro: In the PMGRC study, we are interested in doing different kinds of genetic testing, some that is not available to your doctors regularly, to look for changes that might explain your health concerns

---

Node ID: Zgg7vB9

Parents: 8Z6qtgu

Children: iCSWyTS

User Transcript: Great

Node ID: iCSWyTS

Parents: Zgg7vB9

Children: GLzHadvB

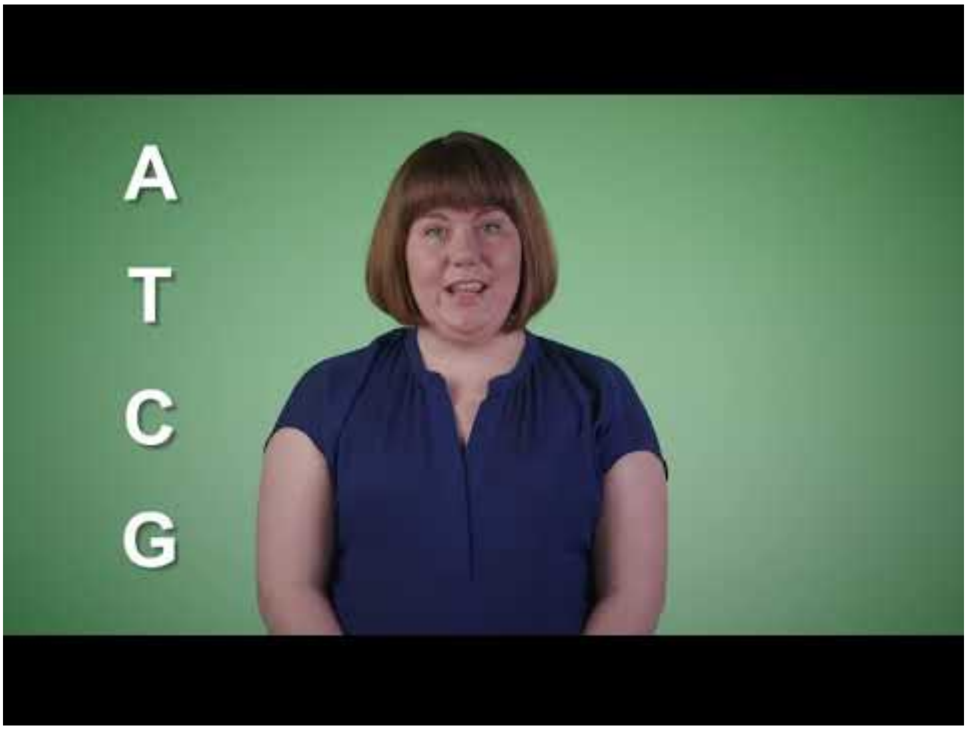

Kauro: Video link: https://www.youtube-nocookie.com/embed/jhJW69Ols14

Kauro: Here's a 1-minute video that talks about one common type of testing, called sequencing

---

Node ID: GLzHadvB

Parents: iCSWyTS

Children: jczufDn

User Transcript: Interesting

Node ID: jczufDn

Parents: iCSWyTS, GLzHadvB

Children: GLzDUvB, 9cuqxwT

Kauro: As we discussed earlier, we are hoping to enroll patients with known or suspected genetic conditions and their blood relatives, such as parents and siblings

Kauro: Can you tell me a little bit more about your history with genetic testing for you or your family members so far?

Node ID: GLzDUvB

Parents: jczufDn

Children: G5dyfh6

User Transcript: Never had genetic testing / I don't recall...

Node ID: G5dyfh6

Parents: GLzDUvB

Children: FiRFHGF

Kauro: No problem!

Kauro: One goal for our PMGRC study is to discover **new** causes of health problems, like finding a new gene or new ways that differences in our genes can cause health concerns

Kauro: If genetic testing didn't find an answer before, our research might be able to find something new

---

Node ID: FiRFHGF

Parents: kYjpLjd, G5dyfh6

Children: SgDDtr8

User Transcript: Okay...

Node ID: SgDDtr8

Parents: FiRFHGF

Children: hxyY83b, LbgZ4YK

Kauro: Another goal of our study is to better understand prior genetic test results that may have been unclear

Kauro: For example, if previous genetic testing found a “variant of uncertain significance”, our research might be able to help explain whether that finding is related to your symptoms or not

---

Node ID: hxyY83b

Parents: SgDDtr8

Children: XKshQvX

User Transcript: Got it!

Node ID: XKshQvX

Parents: DKTqMXc, hxyY83b

Children: oNfduXy

Kauro: We don’t know if this kind of research genetic testing will help people find a diagnosis, but we hope that it will at least help make science and medicine better with the possibility of helping other people in the future

---

Node ID: oNfduXy

Parents: XKshQvX

Children: GokRRmf

User Transcript: Well that sounds nice

Node ID: GokRRmf

Parents: oNfduXy

Children: HEz7sik

Kauro: We want to make sure you know that joining this PMGRC study is **voluntary**

Node ID: HEz7sik

Parents: GokRRmf

Children: NHPvQRe

User Transcript: What does that mean?

Node ID: NHPvQRe

Parents: HEz7sik

Children: Np4Ajah, HUAk3zG, Ua23Tdf

Kauro: Voluntary means you can choose to be part of this study or choose not to be part of this study. If you choose to enroll, then later change your mind, you can withdraw from the study

Kauro: Whatever choice you make, the quality of care you receive from your current medical team will not change. You will not lose access to your medical care or give up any of your legal rights or medical benefits

Kauro: If we make any changes to the study or if there are any important new findings, we will tell you so that you can decide if you still want to participate

---

Node ID: Np4Ajah

Parents: NHPvQRe

Children: ckDLoqi

User Transcript: Sounds good!

Node ID: ckDLoqi

Parents: aPfw9zP, dYrzLWz, Np4Ajah

Children: Z8bgCzz

Kauro: Participating in the study is **free** - so there is no cost to you or to your health insurance provider

Kauro: If you learn something new about your health as a result of the study, you or your insurance provider will be responsible for the cost of any relevant follow-up testing or medical care from those results

---

Node ID: Z8bgCzz

Parents: ckDLoqi

Children: aBzgDD8

User Transcript: Okay

Node ID: aBzgDD8

Parents: Z8bgCzz

Children: ApKCtVL

Kauro: Just a little more background information for you

Kauro: Dr. Eric Vilain is the person responsible for this research study at UCI. This study is paid for by The Human Genome Research Institute (part of the National Institutes of Health or NIH)

Node ID: ApKCtVL

Parents: aBzgDD8

Children: 8XuWfkg, UXhKLb9

Kauro: We are recruiting patients from all over the country, with all kinds of backgrounds and experiences with genetic testing

---

Node ID: 8XuWfkg

Parents: ApKCtVL

Children: M2fEy3b

User Transcript: Makes sense

Node ID: M2fEy3b

Parents: Gs6eCRA, 8XuWfkg

Children: Bq68p3L, HQwx7B3

Kauro: It's important to understand that research testing like this does not replace clinical testing.

Kauro: Whether you join the PMGRC research study or not, you should always continue getting medical advice from your doctor, which might include other options for genetic testing

Kauro: You might want to discuss this study with your family and anyone else you trust before making your decision

---

Node ID: Bq68p3L

Parents: M2fEy3b

Children: 85NXhpr

User Transcript: I definitely will

Node ID: 85NXhpr

Parents: YyMv36M, Bq68p3L

Children: 2GVi3tK

Kauro: So! Let's talk about what will happen when someone enrolls in this study

---

Node ID: 2GVi3tK

Parents: 85NXhpr

Children: FWCoeMN

User Transcript: Yes, please!

Node ID: FWCoeMN

Parents: 2GVi3tK

Children: 7SXetE5

Kauro: At the end of this chat (or after talking to a study team member), if you decide that you do want to join, you will sign an electronic consent form to officially enroll as a participant.

Kauro: Each participant will be assigned a unique study ID number.

Kauro: This unique study ID will be used to label your study samples and data (instead of using your name or other identifying information)

Node ID: 7SXetE5

Parents: FWCoeMN

Children: LUuSmzi

User Transcript: That's reassuring

Node ID: LUuSmzi

Parents: 7SXetE5

Children: dM5aFtN, XxZNtXo, 6wCXYUp

Kauro: Once enrolled, you will be asked to provide some medical information and a detailed family health history

Kauro: The medical information we collect may include things like doctors' notes, current or previous diagnoses, and past test results (both genetic and non-genetic)

Kauro: If you had previous genetic testing (such as whole genome sequencing), you may be asked to provide the data from this testing

---

Node ID: dM5aFtN

Parents: LUuSmzi

Children: i83McKa

User Transcript: Sounds easy enough

Node ID: i83McKa

Parents: dM5aFtN, eX5Kmqt, AJ5y8rf

Children: hcVjm7T

Kauro: Please be aware that the information we collect will not be provided back to you directly

---

Node ID: hcVjm7T

Parents: i83McKa

Children: Af3kSyQ

User Transcript: Okay

Node ID: Af3kSyQ

Parents: hcVjm7T

Children: kmCfuJA, 832xEwo, i6bA6Jp

Kauro: Once enrolled, you may be asked to provide a tissue sample for genetic testing. This sample could be blood, saliva (spit), or cheek swab (also called “buccal swab”).

Kauro: However, not every PMGRC participant will need to provide a sample

---

Node ID: kmCfuJA

Parents: Af3kSyQ

Children: QdRbbvT

User Transcript: I understand

Node ID: QdRbbvT

Parents: is9TJxK, 9aApxUq, kmCfuJA

Children: jo5VEXs

Kauro: And that’s pretty much it! There are no other required interactions

---

Node ID: jo5VEXs

Parents: QdRbbvT

Children: Fok52cR

User Transcript: So what happens next?

Node ID: Fok52cR

Parents: jo5VEXs

Children: DQsLsQj, dFniP5t

Kauro: Once you’ve enrolled and provided the requested information and/or samples, a team of study investigators will review your case history and genetic results

Kauro: This will help them decide what research tests will be performed on your samples and data

---

Node ID: DQsLsQj

Parents: Fok52cR

Children: 2DC2FU2

User Transcript: That’s easy!

Node ID: 2DC2FU2

Parents: dJioePa, DQsLsQj

Children: fjogsGG

Kauro: Our PMGRC study staff might contact you to request additional samples or more information from you

---

Node ID: fjogsGG

Parents: 2DC2FU2

Children: 53ZCpC3

User Transcript: What if I don't want to provide additional samples or information?

Node ID: 53ZCpC3

Parents: fjogsGG

Children: P32EkeA

Kauro: You can decline these additional requests at any time and still be part of the study

---

Node ID: P32EkeA

Parents: 53ZCpC3

Children: 2kEyysx

User Transcript: Who has access to the study information?

Node ID: 2kEyysx

Parents: P32EkeA

Children: SS9NdGi

Kauro: As we mentioned before, the samples and data we gather from you will be labeled only with your unique study ID number. This means that your data and samples are de-identified

Kauro: Only a few members of the study team will be able to link your study number to your name

Kauro: Other collaborators (such as a commercial lab or a mobile blood draw company) would only have your name and identifiers if you used that company for previous genetic testing or blood collection

---

Node ID: SS9NdGi

Parents: 2kEyysx

Children: dZRNUod

User Transcript: That's good to know

Node ID: dZRNUod

Parents: SS9NdGi

Children: 4hnYNnR

Kauro: Our PMGRC study is one of 5 Genomics Research Centers. We collaborate with the other 4 centers and might share your genetic information and/or health information with their experts. Any information we share will be de-identified, so these centers will not have access to your name or other personal information that directly identifies you

Kauro: This study will also send de-identified data to a National Institutes of Health (NIH)-designated data repository. This repository may be accessed by other researchers around the world

---

Node ID: 4hnYNnR

Parents: dZRNUod

Children: RqztaTu

User Transcript: Could my information be used anywhere else?

Node ID: RqztaTu

Parents: 4hnYNnR

Children: Nki2gNX

Kauro: The results of this research may be presented at scientific meetings or in scientific publications

Kauro: You will not be personally identified and no identifiable photos will be published without your specific consent

---

Node ID: Nki2gNX

Parents: RqztaTu

Children: 5vshPHV

User Transcript: Okay, that's good to know

Node ID: 5vshPHV

Parents: Nki2gNX

Children: UH32G43

Kauro: Your de-identified **genetic information** and **health information** could be used for future research studies or given to another investigator for future research studies without your additional informed consent.

Kauro: This de-identified information may be used for many different research studies, for many years in the future.

Kauro: If you enroll in the study, you will have the option to let us know if you want to allow your **samples** to be used for other research beyond this study.

Kauro: Otherwise, your samples will remain at UCI for use in this study only

---

Node ID: UH32G43

Parents: 5vshPHV

Children: W6x9XW5

User Transcript: Good to know!

Node ID: W6x9XW5

Parents: UH32G43

Children: ieCqQsR

Kauro: So far we've been talking about the ways that de-identified information may be used or shared, but you might be wondering whether identifiable information will ever be shared.

Kauro: Your identifiable personal information will not be given to anyone else unless you give your permission in writing first, with a few exceptions that I will explain in a moment.

Kauro: The study team is careful with the information we gather for the study, and we will only share it with authorized members of the study team or people who need to review the study information.

Node ID: ieCqQsR

Parents: W6x9XW5

Children: 5aJHekM

User Transcript: Who might have a need to review the study information?

Node ID: 5aJHekM

Parents: ieCqQsR

Children: 4pcbkEp

Kauro: There are some third parties such as government agencies or other groups within UCI that may check records that identify you without your permission

Kauro: They might look at the study records and your medical records to make sure we are following the law and protecting the people in the study and to make sure our study results are correct

Kauro: Some agencies or groups who might see these records are:

- The Department of Health and Human Services Office of Human Research Protections
- The National Human Genome Research Institute (part of the NIH)
- The UCI Medical Center Institutional Review Board (the ethics board that reviewed and approved this research study)
- The Office for the Protection of Human Subjects

Node ID: 4pcbkEp

Parents: 5aJHekM

Children: 4SVWLG2

User Transcript: Okay, that makes sense

Node ID: 4SVWLG2

Parents: 4SVWLG2

Children: eq8AgVx

Kauro: You may have heard of HIPAA before, but we want to make sure you know the details and how it applies to your participation in this study

---

Node ID: eq8AgVx

Parents: 4SVWLG2

Children: 3WrbYFW

User Transcript: Isn't that something I sign at my doctor's office?

Node ID: 3WrbYFW

Parents: eq8AgVx

Children: DB7z6ev

Kauro: Yes, you have probably reviewed the HIPAA information at medical visits

Kauro: HIPAA is a privacy law that protects your individually identifiable health information (also called "Protected Health Information" or "PHI")

Kauro: The **HIPAA** law also describes how information about you may be used or shared if you are in a research study

---

Node ID: DB7z6ev

Parents: 3WrbYFW

Children: QWBbjLo

User Transcript: That makes sense

Node ID: QWBbjLo

Parents: DB7z6ev

Children: MpZMNKu

Kauro: Under HIPAA, you need to sign an agreement in order for researchers to use or share your PHI for research purposes

Kauro: I can provide you with a link to UCI's HIPAA Information Sheet at the end of our chat. You can read the complete HIPAA authorization in the consent document

---

Node ID: MpZMNKu

Parents: QWBbjLo

Children: 63uUQep

User Transcript: 👍

Node ID: 63uUQep

Parents: MpZMNKu

Children: hqiSxfQ

Kauro: It is important that you read this carefully and ask a member of the research team to explain anything you do not understand

---

Node ID: hqiSxfQ

Parents: 63uUQep

Children: bTii8uW

User Transcript: Can you guarantee that my information will be kept private?

Node ID: bTii8uW

Parents: hqiSxfQ

Children: fyZsjfV, NP5jatP

Kauro: We will make every effort to keep your information private, but no one's privacy can be totally guaranteed

Kauro: To help protect your privacy, the research team has obtained a **Certificate of Confidentiality** from the Department of Health and Human Services (DHHS)

Kauro: With this certificate, the investigators cannot be forced (for example, by a court order or subpoena) to give information that may identify you in any federal, state or local civil or criminal court, or in any administrative, legislative, or other proceeding

---

Node ID: fyZsjfV

Parents: bTii8uW

Children: cgwAN3K

User Transcript: Good to know

Node ID: cgwAN3K

Parents: QndKtpj, fyZsjfV

Children: coJCK6d

Kauro: Let's talk about what types of results this research could find

---

Node ID: coJCK6d

Parents: cgwAN3K

Children: UfYVcme

User Transcript: Yes!

Node ID: UfYVcme

Parents: coJCK6d

Children: AAoupYe

Kauro: If your samples and/or data are studied as part of this research, there are different types of results that could be returned to you

Kauro: Like we mentioned earlier, you are being referred to this study because you or your family member has a known or suspected genetic condition based on specific health concerns or symptoms

---

Node ID: AAoupYe

Parents: UfYVcme

Children: L82MnJD

User Transcript: Right

Node ID: L82MnJD

Parents: AAoupYe

Children: 9btMpEf

Kauro: This PMGRC study will try to find genetic changes that may be related to those issues

Kauro: These results are called **primary findings**, and they often allow the team to make a diagnosis

Kauro: Later in this chat, you will get to choose whether you want to be told about these primary findings

---

Node ID: 9btMpEf

Parents: L82MnJD

Children: YJ9BvJu

User Transcript: Great!

Node ID: YJ9BvJu

Parents: 9btMpEf

Children: XW2AGZ2

Kauro: Sometimes the study may find genetic variants that are not related to the patient’s current known health concerns

Kauro: These results are called **secondary findings**. Secondary findings are considered **medically actionable**, meaning if you are found to have one of these findings on your testing, then you or your healthcare provider can take action to reduce your risk of developing the health concerns or to better treat or manage the health concerns

---

Node ID: XW2AGZ2

Parents: YJ9BvJu

Children: PLHGemU

User Transcript: What kind of findings are medically actionable?

Node ID: PLHGemU

Parents: XW2AGZ2

Children: mC7SWtw

Kauro: Some examples of medically actionable secondary findings may be genetic variants that cause increased risk for things like heart disease or cancer

Kauro: Secondary findings are often related to adult-onset health risks, but it is possible that we could discover a secondary finding that relates to health issues in childhood

---

Node ID: mC7SWtw

Parents: PLHGemU

Children: Et3pvov

User Transcript: Okay, I understand

Node ID: Et3pvov

Parents: mC7SWtw

Children: Py4Sw9s

Kauro: Knowing about a medically actionable genetic variant can help your doctors to adjust your medical care plan

Kauro: This can include things like avoiding certain medications or having more frequent checkups with your doctor

---

Node ID: Py4Sw9s

Parents: Et3pvov

Children: Qakavzg

User Transcript: That’s good to know

Node ID: Qakavzg

Parents: Py4Sw9s

Children: n2xeLXt

Kauro: The study could also discover secondary findings that are **not medically actionable**, meaning they could be related to an increased risk for certain diseases with no known treatments or interventions

---

Node ID: n2xeLXt

Parents: Qakavzg

Children: X5VskxR

User Transcript: I'll keep that in mind

Node ID: X5VskxR

Parents: n2xeLXt

Children: 78Ucru5

Kauro: It's important to note that our research team will not be analyzing every sample for secondary findings, but it is possible that we will identify secondary findings in the course of the study

---

Node ID: 78Ucru5

Parents: X5VskxR

Children: NETsCwx

User Transcript: What if I don't want to know about health risks from secondary findings?

Node ID: NETsCwx

Parents: 78Ucru5

Children: DqZpVap

Kauro: That's perfectly fine

Kauro: If you do decide to participate in the study, you will get to decide if you want to receive any possible research results. You will decide if you want to receive everything or receive only some types of results and not others

---

Node ID: DqZpVap

Parents: NETsCwx

Children: gAkBYPB

User Transcript: That's good to know

Node ID: gAkBYPB

Parents: DqZpVap

Children: gabQsoo

Kauro: Sometimes genetic testing can find unexpected or surprising results that aren't related to your health

Kauro: For example, sometimes testing can reveal that a child's parent is not actually their biological parent or that two parents are related to each other by blood

Kauro: These types of unexpected results are called **incidental findings**

Node ID: gabQsoo

Parents: gAkBYPB

Children: aYfinQF

User Transcript: Oh, okay

Node ID: aYfinQF

Parents: gabQsoo

Children: PF2Sqz8

Kauro: We do not plan to go looking for these types of incidental findings and we don't plan to tell you about them

Kauro: But it is possible that you could figure out these incidental results based on other genetic test results we may share with you

---

Node ID: PF2Sqz8

Parents: aYfinQF

Children: gXJJXUo

User Transcript: I understand

Node ID: gXJJXUo

Parents: PF2Sqz8

Children: RSiG59t, gEQnvho

Kauro: If the study team finds a genetic change that relates to your health concerns, we will tell you about it if you have opted to receive that kind of research result

Kauro: Because the results from our study are research results and not clinical results, we will not provide you with an official clinical report

Kauro: Any research findings will need to be **confirmed in a certified clinical lab** before they can be added to your medical record

---

Node ID: RSiG59t

Parents: gXJJXUo, LUkpmdJ

Children: 2MNY2vm

User Transcript: Who will pay for that additional testing?

Node ID: 2MNY2vm

Parents: RSiG59t

Children: kkG2ixC, gEQnvho

Kauro: You or your insurance company will have to pay for those additional services

---

Node ID: kkG2ixC

Parents: 2MNY2vm

Children: EJkzrsP

User Transcript: Got it

Node ID: EJkzrsP

Parents: YjX3aRS, kkG2ixC

Children: Y3WMAjr

Kauro: Just so you know, we expect this PMGRC study to last at least until 2026, but you can choose to stop participating at any time

Kauro: After signing the Consent/Authorization, you can change your mind and either revoke or withdraw your authorization in the future

---

Node ID: Y3WMAjr

Parents: EJkzrsP

Children: AbjKtPR

User Transcript: Good to know!

Node ID: AbjKtPR

Parents: Y3WMAjr

Children: MamuHnC

Kauro: To revoke the authorization, you must send a written letter to the Principal Investigator to inform him of your decision

Kauro: Eric Vilain, MD, PhD
University of California, Irvine (UCI)
Department of Pediatrics
1003 Health Sciences Rd, Suite 308
Irvine, CA 92617

Kauro: If you revoke this Authorization, researchers may only use and disclose the PHI that was collected for this research study before you revoked the authorization

Kauro: If you change your mind and withdraw the authorization, you will not be allowed to participate in the study any further

---

Node ID: MamuHnC

Parents: AbjKtPR

Children: GKg8Z3M

User Transcript: I understand

Node ID: GKg8Z3M

Parents: MamuHnC

Children: jdAqu9q

Kauro: It is important that you know that anyone who undergoes genetic testing in the United States is protected from certain types of discrimination by a policy called the **Genetic Information Nondiscrimination Act (GINA)**

Node ID: jdAqu9q

Parents: GKg8Z3M

Children: VdJaPdJ

User Transcript: What areas are protected?

Node ID: VdJaPdJ

Parents: jdAqu9q

Children: HE7Z96Y, JkATxFN, Hyni8Jx

Kauro: Generally, GINA makes it illegal for health insurance companies, group health insurance plans, and most employers to discriminate against you based on your genetic information

Kauro: However, this does not apply to companies with fewer than 15 employees or the United States military

---

Node ID: HE7Z96Y

Parents: VdJaPdJ, JHvZPbt

Children: iyPt6Ye

User Transcript: How is health insurance protected?

Node ID: iyPt6Ye

Parents: HE7Z96Y

Children: K9gS6Lu, JkATxFN

Kauro: Health insurance companies may not request your genetic information that we get from this research

Kauro: Health insurance companies may not use your genetic information when deciding whether to insure you or the amount of money they will charge for your plan

---

Node ID: K9gS6Lu

Parents: iyPt6Ye

Children: RpJXYSM

User Transcript: I understand

Node ID: RpJXYSM

Parents: HLbqQro, K9gS6Lu, Hyni8Jx

Children: EvQQVDh, abqe8JH, htJY7WJ

Kauro: It's also very important to know that GINA does not protect you against genetic discrimination by companies that sell life insurance, disability insurance, or long-term care insurance

---

Node ID: EvQQVDh

Parents: RpJXYSM

Children: nk6nfNp

User Transcript: What does that mean?

Node ID: nk6nfNp

Parents: EvQQVDh

Children: JQkEcaY

Kauro: These kinds of additional insurance companies (such as life insurance or disability insurance) might ask you to provide your medical history–including genetic test results–before allowing you to enroll

Kauro: Because the genetic testing being done in this study is for research only, the results will not be part of any official medical record until they are confirmed by a clinical lab.

Kauro: Some families may choose to enroll in these kinds of insurances for their child or themselves before getting a clinically validated genetic test result

---

Node ID: JQkEcaY

Parents: nk6nfNp

Children: 8NYKg4h

User Transcript: Okay

Node ID: 8NYKg4h

Parents: M8MdRDQ, JQkEcaY, htJY7WJ

Children: 9s6U2mp

Kauro: I'd like to take a moment to give you some info about the **risks and possible discomforts** that may occur as part of participating in this study

Kauro: The greatest risks of the study are related to sample collection, the potential for loss of confidentiality of your study information, and possible psychological risks associated with genetic information

Kauro: That may sound like a lot, so I will go through each of these with you

---

Node ID: 9s6U2mp

Parents: 8NYKg4h

Children: Co5pFGN

User Transcript: Okay, Kauro 👍

Node ID: Co5pFGN

Parents: 9s6U2mp

Children: DhAQkSy

Kauro: There are some risks related to taking the blood sample, such as discomfort and/or bruising

Kauro: Infection, excess bleeding, clotting, or fainting can happen

Kauro: There may be minimal discomfort in the cheek if a cheek swab sample is obtained

---

Node ID: DhAQkSy

Parents: Co5pFGN

Children: R87ndyg

User Transcript: Okay, I understand

Node ID: R87ndyg

Parents: DhAQkSy

Children: YcJnDbx

Kauro: The study team will take care to protect the privacy and confidentiality of your study data

Kauro: Because your samples and data will be labeled only with a study ID, your personal information (like your name) will only be known to certain authorized study team members

Kauro: The team will keep your personal and genetic data in an encrypted secure environment, and be notified immediately if there is a security breach

---

Node ID: YcJnDbx

Parents: R87ndyg

Children: Mz69DPK

User Transcript: That's good to know

Node ID: Mz69DPK

Parents: YcJnDbx

Children: UTaGRW8, YDBEbUp

Kauro: However, even though your samples and data will not be labeled with your name or other personal information, your genetic information is unique to you

Kauro: So it is possible that someone could identify you from your genetic information or learn something about you by looking at your genetic information

Kauro: This risk may increase in the future as technologies advance and as more researchers study your genetic information

---

Node ID: UTaGRW8

Parents: Mz69DPK

Children: fsbFZtR

User Transcript: Ah, I see

Node ID: fsbFZtR

Parents: YDBEbUp, UTaGRW8

Children: D8z5whK

Kauro: These risks may also affect members of your family. You may want to discuss your participation in this study with your family and ask the study team about any questions or concerns you may have

---

Node ID: D8z5whK

Parents: fsbFZtR

Children: cMmQUhN

User Transcript: Okay, I'll keep that in mind

Node ID: cMmQUhN

Parents: D8z5whK

Children: R6TFjLi

Kauro: There can also be some risks associated with learning about genetic results that may be discovered through this study

Kauro: Genetic testing can sometimes reveal results that you were not expecting or even things you do not want to know

Kauro: It is possible that learning about your genetic results may make you feel distressed or anxious

---

Node ID: R6TFjLi

Parents: cMmQUhN

Children: KbzEchg

User Transcript: Ah, okay

Node ID: KbzEchg

Parents: R6TFjLi

Children: 3uRFDJg

Kauro: These results may be more certain than others

Kauro: For example, as we discussed before, we may find a “variant of uncertain significance”, which means that we aren’t sure whether a genetic variant is linked to a specific health concern or not

Kauro: Some people may feel anxious or uncomfortable about uncertain results

---

Node ID: 3uRFDJg

Parents: KbzEchg

Children: d4nnpdr

User Transcript: Okay

Node ID: d4nnpdr

Parents: 3uRFDJg

Children: 9hePugt

Kauro: Lastly, results from this study may sometimes give you information about a genetic health issue that does not have a clear medical treatment

Kauro: Some people become distressed or anxious to learn about a diagnosis or risk to develop symptoms without a known treatment

Kauro: It is up to you to decide if you want to receive any possible genetic results from this study. As we discussed before, you can decide if you want to receive some types of results but not others

---

Node ID: 9hePugt

Parents: d4nnpdr

Children: KX6JCsF

User Transcript: That’s good to know

Node ID: KX6JCsF

Parents: 9hePugt

Children: jey9scg

Kauro: Lets also talk about the **possible benefits** of participating in this study

Kauro: You should know that there may be no benefit to you for your participation in this study, and you will not be paid for your participation in this study

---

Node ID: jey9scg

Parents: KX6JCsF

Children: h4Luo4M

User Transcript: Okay, I understand

Node ID: h4Luo4M

Parents: jey9scg

Children: RsJ5kCQ, UynEV5h

Kauro: Some study participants may benefit by receiving a new genetic diagnosis or discovering the genetic cause of a known genetic health concern

Kauro: This type of information is beneficial because it can help that individual understand more about their health, provide their clinical team some guidance about testing or screening options, and in rare cases, can even help the individual learn about a specific therapy or treatment

---

Node ID: RsJ5kCQ

Parents: h4Luo4M

Children: 7WLks8j

User Transcript: Okay, that makes sense

Node ID: 7WLks8j

Parents: UynEV5h, RsJ5kCQ

Children: KuX3ekD

Kauro: Unfortunately, for many people who have a suspected genetic health concern, a specific diagnosis or cause may never be found

Kauro: We do not know if the genetic testing used in this study will be able to help diagnose people who do not yet have a diagnosis

Kauro: We hope that through this study we will learn new information and improve our understanding of genetic conditions to help us find diagnoses for people in the future

---

Node ID: KuX3ekD

Parents: 7WLks8j

Children: nYueqrk

User Transcript: Okay

Node ID: nYueqrk

Parents: KuX3ekD

Children: 5UhK2Hy

Kauro: I'm going to give you some contact information, and you can write it down if you would like. ✏️

Kauro: If you have any questions about the study or if you get hurt or sick because of something to do with the study, please contact the principal investigator, Dr. Eric Vilain, at 949-824-4090.

Node ID: 5UhK2Hy

Parents: nYueqrk

Children: BXUr8TN

User Transcript: Thanks for this information

Node ID: BXUr8TN

Parents: 5UhK2Hy

Children: QvCduQU

Kauro: If you have any questions or concerns about your privacy rights, you may contact the UCI Health Privacy Office at 714-456-3916

Kauro: If you have questions or concerns about your rights as a study participant, you can contact UCI's Human Research Protections unit in the Office of Research at 949-824-6068, or by email (IRB@research.uci.edu), or by writing to us at 160 Aldrich Hall, Irvine, CA 92697

Node ID: QvCduQU

Parents: BXUr8TN

Children: Dq4hG9k

User Transcript: Okay

Node ID: Dq4hG9k

Parents: QvCduQU

Children: BKUeKkp, FVdUisi, NsfXTD7, BMeYncH

Kauro: Whew! 😅 We just went through a lot of information together

Kauro: I've gone through all the topics I need to cover

Kauro: Now, it's up to you to decide if you would like to give your consent for yourself or your child to participate in the PMGRC study

---

Node ID: BKUeKkp

Parents: Dq4hG9k, h9rpY7K, ixSPowJ

Children: SZabuK4

User Transcript: I am ready to consent!

Node ID: SZabuK4

Parents: BKUeKkp

Children: bmorWZo

Kauro: Okay, great 😀

Kauro: In a moment, I will ask you to make some choices about how you want to participate

Kauro: But first, I need to ask you some questions to make sure you understand the basics of participating in the PMGRC study

Kauro: And don't worry - if you answer any of these questions incorrectly, I'll help remind you of the right answer

---

Node ID: bmorWZo

Parents: SZabuK4

Children: VWzWNAK

User Transcript: Okay - I'm ready

Node ID: VUipQHn

Parents: nFpTPVg

Children: JTCaWss

Kauro: You got 10 out of 10 questions right! 🥇

Kauro: Now we're ready to collect your consent for participation in the study

Kauro: We'll start by capturing your enrollment preferences and then we'll ask for your electronic signature

---

Node ID: QKNbLGv

Parents: JTCaWss

Children: gdiDJUS

Kauro: We would like to store your research samples in a biobank for future research, which we will describe in a moment

Kauro: The samples we store could include genetic material (DNA or RNA), skin or other tissue samples, or cells

Kauro: The biobank is maintained by the study team at UCI

Kauro: Please let us know how you would like your samples to be used

---

Node ID: gdiDJUS

Parents: QKNbLGv

Children: Psu2bBx

Form ID: sample-storage-use-form

From Text:

Can we store your samples for purposes of this research study? (Required for participation in the study)

Can we store your samples in the UCI biobank for purposes of unrelated research?

From Options:


```
[
  {
    "name": "storeSamplesThisStudy",
    "type": "radio",
    "label": "Study Sample Storage",
    "options": [
      {
        "label": "Yes",
        "value": "yes"
      }
    ]
  },
  {
    "name": "storeSamplesOtherStudies",
    "type": "radio",
    "label": "Other Studies Sample Storage",
    "options": [
      {
        "label": "Yes",
        "value": "yes"
      },
      {
        "label": "No",
        "value": "no"
      }
    ]
  }
]
```

Node ID: Psu2bBx

Parents: gdiDJUS

Children: bdmfVYQ

Kauro: You should know that you may change your mind at a later time and request that your sample be destroyed

Kauro: If you change your mind and want to request that your sample be destroyed, you must send a written request to:

Eric Vilain, MD, PhD
University of California, Irvine (UCI)
Department of Pediatrics
1003 Health Sciences Rd, Suite 308
Irvine, CA 92617

Node ID: bdmfVYQ

Parents: Psu2bBx

Children: cmJfBjf

User Transcript: That’s good to know

Node ID: cmJfBjf

Parents: bdmfVYQ

Children: VZsTdFg

Kauro: We would like to store personal health information (PHI) collected from you in this study (such as your name, date of birth, contact information, etc.) in a database for future research

Kauro: The database is maintained by the study team at UCI

Kauro: Please let us know how you would like your PHI to be stored and used

---

Node ID: VZsTdFg

Parents: cmJfBjf

Children: 4bYChBx

Form ID: phi-use-form

From Text:

Can we store your PHI in our database for research related to this study? (Required for participation in the study)

Can we use your stored PHI to contact you about future research opportunities that are not related to this study?

From Options:

```
[
  {
    "name": "storePhiThisStudy",
    "type": "radio",
    "label": "PHI Storage for Study",
    "options": [
      {
        "label": "Yes",
        "value": "yes"
      }
    ]
  },
  {
    "name": "storePhiOtherStudies",
    "type": "radio",
    "label": "PHI Use for Other Studies",
    "options": [
      {
        "label": "Yes",
        "value": "yes"
      },
      {
        "label": "No",
        "value": "no"
      }
    ]
  }
]
```

Node ID: 4bYChBx

Parents: VZsTdFg

Children: SLcyBjQ, GzG3exY

Kauro: You may recall that we discussed the types of genetic research results that may be found in this study

Kauro: You get to decide which types of results you want to learn about

Kauro: Do you remember the different types of results we discussed, like “primary results” and “medically actionable secondary results”?

Node ID: SLcyBjQ

Parents: 4bYChBx

Children: QpzocPu

User Transcript: Yes, I remember

Node ID: QpzocPu

Parents: hnqNqbi, SLcyBjQ

Children: nriTamw

Kauro: Please complete the form below to let us know what types of results you would like to receive

---

Node ID: nriTamw

Parents: QpzocPu

Children: knbtYck

Form ID: result-return-form

From Text:

From Options:


```
[
  {
    "name": "rorPrimary",
    "type": "radio",
    "label": "Do you want to learn about primary results that are found?",
    "options": [
      {
        "label": "Yes",
        "value": "yes"
      },
      {
        "label": "No",
        "value": "no"
      }
    ]
  },
  {
    "name": "rorSecondary",
    "type": "radio",
    "label": "Do you also want to learn about medically actionable secondary
results?",
    "options": [
      {
        "label": "Yes",
        "value": "yes"
      },
      {
        "label": "No",
        "value": "no"
      }
    ]
  },
  {
```

```
    "name": "rorSecondaryNot",
    "type": "radio",
    "label": "Do you want to learn about secondary results that are NOT
medically actionable?",
    "options": [
      {
        "label": "Yes",
        "value": "yes"
      },
      {
        "label": "No",
        "value": "no"
      }
    ]
  }
]
```

Node ID: knbtYck

Parents: nriTamw

Children: AimWGCA

Kauro: Great! You have now entered all of your choices for your enrollment

Kauro: You are ready to provide your signature to consent!

Node ID: 5n6QHJ7

Parents: AimWGCA

Children: Mzh9KYH

Kauro: Thank you!

Node ID: Mzh9KYH

Parents: nAaeApX, 5n6QHJ7, VbbeudE

Children: KxvpjwE

Kauro: We are almost done with the consent process. Just a few more things to tell you.

Kauro: Below are links to access some of the documents we discussed during our chat:

HIPAA information - https://www.cdc.gov/phlp/publications/topic/hipaa.html

GINA resources - https://www.genome.gov/about-genomics/policy-issues/Genetic-Discrimination#gina

CalGINA information - http://www.leginfo.ca.gov/pub/11-12/bill/sen/sb_0551-0600/sb_559_bill_20110906_chaptered.pdf

---

Node ID: KxvpjwE

Parents: Mzh9KYH, Mzke78A

Children: W7DeBPU

User Transcript: Thank you

Node ID: W7DeBPU

Parents: KxvpjwE

Children: TSRVTj5

Kauro: After our study staff has finalized the consent paperwork, we will send you a copy of the signed consent documents via a secure email using the email address you provided above

Kauro: You will receive a separate email with a transcript of our chat

---

Node ID: TSRVTj5

Parents: W7DeBPU

Children: 39kDMR8

User Transcript: Okay, great!

Node ID: 39kDMR8

Parents: TSRVTj5

Children: 6iruWjc

Kauro: We will also send you an email with a link to an online form where we will collect health information to complete your study enrollment

Kauro: Once you are enrolled, we will be in touch to let you know if we need to collect any samples from you or your child

---

Node ID: 6iruWjc

Parents: 39kDMR8

Children: nJgsH7y

User Transcript: That sounds good

Node ID: nJgsH7y

Parents: nqDwZrd, 6iruWjc, MNpRjAA, PZYk7Ga

Children: BUgU8Tq, nTBdMds

Kauro: Remember, if you have any questions or concerns, you can always reach out to our team at GREGOR@hs.uci.edu

Kauro: If you would like to help me improve, please rate your experience and provide feedback!

Node ID: BUgU8Tq

Parents: nJgsH7y

Children: YQH834A, aXDDbh2

User Transcript: I'd like to leave feedback

Node ID: YQH834A

Parents: BUgU8Tq

Children: aXDDbh2

Kauro: Great, thanks so much!

Node ID: aXDDbh2

Parents: BUgU8Tq, YQH834A

Children: YQHvnrA

Form ID: user-feedback-form

From Text:

From Options:


```
[
  {
    "name": "satisfaction",
    "type": "select",
    "label": "Overall satisfaction with the consent process",
    "options": [
      {
        "label": "Very Satisfied",
        "value": "Very Satisfied"
      },
      {
        "label": "Satisfied",
        "value": "Satisfied"
      },
      {
        "label": "Neutral",
        "value": "Neutral"
      },
      {
        "label": "Dissatisfied",
        "value": "Dissatisfied"
      },
```

```
      {
        "label": "Very Dissatisfied",
        "value": "Very Dissatisfied"
      }
    ],
    "required": true
  },
  {
    "name": "suggestions",
    "type": "textarea",
    "label": "Suggestions for Improvement"
  }
]
```

Node ID: nTBdMds

Parents: nJgsH7y

Children: 7PxxtTh

User Transcript: No thanks

Node ID: GzG3exY

Parents: 4bYChBx

Children: 3iBfwGK

User Transcript: No, can you remind me?

Node ID: 3iBfwGK

Parents: GzG3exY

Children: WHwSvgC

Kauro: Primary findings are genetic changes that may be related to the specific health concern that has been seen in you or your family members

Kauro: Primary findings often allow the clinical team to make a diagnosis

---

Node ID: WHwSvgC

Parents: 3iBfwGK

Children: N9JZqfY

User Transcript: Okay

Node ID: N9JZqfY

Parents: WHwSvgC

Children: Hc525XA

Kauro: Sometimes the study may find genetic variants that are not related to a patient's current known health concerns

Kauro: These are called **secondary findings**

Kauro: Some of these secondary findings are considered medically actionable, meaning if these genetic changes are known, then you or your healthcare provider can then take action to try to reduce your risk of developing the symptoms or to better treat or manage your healthcare related to the secondary findings

---

Node ID: Hc525XA

Parents: N9JZqfY

Children: GMm2FJg

User Transcript: What kind of findings are medically actionable?

Node ID: GMm2FJg

Parents: Hc525XA

Children: hnqNqbi

Kauro: Some examples of medically actionable secondary findings may be genetic variants that cause increased risk for future health concerns like heart disease or cancer

Kauro: Secondary findings are often related to adult-onset health risks. However, it is possible that we could discover a secondary finding that relates to symptoms in childhood

---

Node ID: hnqNqbi

Parents: GMm2FJg

Children: QpzocPu

User Transcript: Okay

Node ID: nbkJ3g7

Parents: JTCaWss

Children: YkoAEVe

Kauro: Now, let's talk about the next steps for enrolling your child or children

Kauro: How many children are you enrolling?

Node ID: YkoAEVe

Parents: nbkJ3g7

Children: eDqsGdn, VbbeudE

Form ID: num-children-enroll-form

From Text:

From Options:


```
                                                                    [
  {
    "name": "numChildrenEnroll",
    "type": "radio",
    "label": "How many children are you enrolling?",
    "options": [
      {
        "label": "1",
        "value": "1"
      },
      {
        "label": "2",
        "value": "2"
      },
      {
        "label": "3",
        "value": "3"
      },
      {
        "label": "4 or more",
        "value": "4"
      }
    ]
  }
]
```

Node ID: Mzke78A

Parents: nAaeApX

Children: KxvpjwE

Kauro: We are almost done with the consent process. Just a few more things to tell you.

Kauro: If you enrolled a child who is 7 years or older, a member of the study team will need to talk to your child briefly to tell them about the study and confirm their interest in participating

Kauro: Below are links to access some of the documents we discussed during our chat:

HIPAA information - https://www.cdc.gov/phlp/publications/topic/hipaa.html

GINA resources - https://www.genome.gov/about-genomics/policy-issues/Genetic-Discrimination#gina

CalGINA information - http://www.leginfo.ca.gov/pub/11-12/bill/sen/sb_0551-0600/sb_559_bill_20110906_chaptered.pdf

---

Node ID: VbbeudE

Parents: YkoAEVe

Children: Mzh9KYH

Kauro: Okay, great!

Kauro: To enroll four or more children, it would be best for you to talk to a member of our study team

Kauro: Someone from our team will contact you directly by phone or email within the next 2-3 weeks

Kauro: If you have questions now, you can contact us at GREGOR@hs.uci.edu

---

Node ID: G3EXSPz

Parents: nFpTPVg

Children: DdxFUae, kZ6qj4C

Kauro: It seems like you had a little trouble with these questions

Kauro: I hope my responses helped clarify.

Kauro: Would you like to go through them one more time, or would you like to talk to a member of our study team?

Node ID: nqDwZrd

Parents: DdxFUae

Children: nJgsH7y

Kauro: Sure thing

Kauro: Someone from our study team will contact you directly by phone or email within the next 2-3 weeks

---

Node ID: iH6N9fF

Parents: nFpTPVg

Children: X4CvZHZ

Kauro: I am sorry. You did not get all the answers correct.

Kauro: To complete your consent, we'd like to connect you to a member of our study team

---

Node ID: PZYk7Ga

Parents: X4CvZHZ

Children: nJgsH7y

Kauro: Someone from our team will contact you directly by phone or email within the next 2-3 weeks

---

Node ID: Swxbt6c

Parents: FVdUisi

Children: MNpRjAA

Kauro: Okay - that's just fine

Node ID: MNpRjAA

Parents: Swxbt6c

Children: nJgsH7y

User Transcript: Okay, sounds good!

Node ID: P8C5vVo

Parents: NsfXTD7

Children: kiRj7cx

Kauro: Okay! Please take all the time you need

Kauro: As you are considering things, you can scroll through our chat to review our discussion any time. To come back to the chat, click the link you used to access the chat

Kauro: If the link is expired or does not work, the study team can send a new link

---

Node ID: kiRj7cx

Parents: P8C5vVo

Children: ixSPowJ

User Transcript: Sounds good

Node ID: ixSPowJ

Parents: kiRj7cx

Children: BKUeKkp, FVdUisi

Kauro: When you are ready to decide, you can come back and continue this chat

---

Node ID: h9rpY7K

Parents: BMeYncH

Children: BKUeKkp, FVdUisi

Kauro: Okay! I can arrange for someone from our study team to contact you directly by phone and/ or email within the next 2-3 weeks

Kauro: If you have questions now, you can email us at GREGOR@hs.uci.edu

Kauro: You can also come back here to continue the chat if you have made a decision

---

Node ID: UynEV5h

Parents: h4Luo4M

Children: 7WLks8j

User Transcript: That would be great

Node ID: YDBEbUp

Parents: Mz69DPK

Children: fsbFZtR

User Transcript: That is concerning

Node ID: abqe8JH

Parents: RpJXYSM

Children: nB4pVm8

User Transcript: Do I need to worry about this?

Node ID: nB4pVm8

Parents: abqe8JH

Children: M8MdRDQ

Kauro: Because the genetic testing being done in this study is for research only, the results will not be part of any official medical record until they are confirmed by a clinical lab

Kauro: Some families may choose to enroll in these kinds of insurances for their child or themselves before getting a clinically-validated genetic test result

---

Node ID: M8MdRDQ

Parents: nB4pVm8

Children: 8NYKg4h

User Transcript: Okay

Node ID: htJY7WJ

Parents: RpJXYSM

Children: 8NYKg4h

User Transcript: Okay

Node ID: JkATxFN

Parents: VdJaPdJ, iyPt6Ye

Children: JHvZPbt

User Transcript: How are employers involved?

Node ID: JHvZPbt

Parents: JkATxFN

Children: HLbqQro, HE7Z96Y

Kauro: Employers may not use your genetic information that we get from this research when deciding to hire, promote, or fire you

---

Node ID: HLbqQro

Parents: JHvZPbt

Children: RpJXYSM

User Transcript: I understand

Node ID: Hyni8Jx

Parents: VdJaPdJ

Children: RpJXYSM

User Transcript: I understand

Node ID: gEQnvho

Parents: gXJJXUo, 2MNY2vm

Children: LUkpmdJ

User Transcript: What does "confirmed in a clinical lab" mean?

Node ID: LUkpmdJ

Parents: gEQnvho

Children: YjX3aRS, RSiG59t

Kauro: Most tests done on samples in research studies are **only for research** and have no clear meaning for healthcare

Kauro: If we think that there are research results that might have meaning for you or your family, these tests should be re-done by a certified clinical laboratory to confirm them

Kauro: Results will only be placed in your medical record once they have been clinically confirmed

---

Node ID: YjX3aRS

Parents: LUkpmdJ

Children: EJkzrsP

User Transcript: I understand

Node ID: NP5jatP

Parents: bTii8uW

Children: 9SsWRKf

User Transcript: What if I tell people I’m part of this study?

Node ID: 9SsWRKf

Parents: NP5jatP

Children: QndKtpj

Kauro: It is important that you know that a Certificate of Confidentiality does not stop you or a member of your family from voluntarily giving information to others about you or about taking part in this research

Kauro: You should also know that if an insurer or employer learns about your participation and you give them permission to receive research information about you, we cannot use the Certificate of Confidentiality to keep your information private from them

Kauro: This means that you must also actively protect your own privacy

---

Node ID: QndKtpj

Parents: 9SsWRKf

Children: cgwAN3K

User Transcript: Got it

Node ID: dFniP5t

Parents: Fok52cR

Children: dJioePa

User Transcript: What kinds of research tests?

Node ID: dJioePa

Parents: dFniP5t

Children: 2DC2FU2

Kauro: Your sample may be used for a variety of genetic testing including, but not limited to: Whole Genome Sequencing, Optical Mapping, and RNA sequencing.

Kauro: These are different ways of looking at your genetic material

---

Node ID: 832xEwo

Parents: Af3kSyQ, CFMdbEP

Children: jzxix3y

User Transcript: Where will my blood be drawn?

Node ID: jzxix3y

Parents: 832xEwo

Children: 9aApxUq, i6bA6Jp

Kauro: There are a few options

Kauro: If you are having clinical blood drawn for another reason, an extra sample may be collected for this study at that time

Kauro: You could also schedule a blood draw at the UCI research unit or ask if our study team is able to arrange for a mobile phlebotomy company to come to your home for a blood draw

---

Node ID: 9aApxUq

Parents: jzxix3y

Children: QdRbbvT

User Transcript: So there are options. Got it

Node ID: i6bA6Jp

Parents: Af3kSyQ, jzxix3y

Children: CFMdbEP

User Transcript: What’s a buccal swab?

Node ID: CFMdbEP

Parents: i6bA6Jp

Children: is9TJxK, 832xEwo

Kauro: A “buccal swab” is like a large Q-tip used for swabbing the inside of the cheeks.

Kauro: If you provide a buccal sample, these will be collected at home with a kit provided to you with instructions for collection at home and mailing back. Sometimes your doctor at UCI can help you collect your buccal swab

---

Node ID: is9TJxK

Parents: CFMdbEP

Children: QdRbbvT

User Transcript: Cool!

Node ID: XxZNtXo

Parents: LUuSmzi

Children: nUA9owA

User Transcript: Where do I get my doctor’s notes?

Node ID: nUA9owA

Parents: XxZNtXo, SvnYgFC

Children: AJ5y8rf, 6wCXYUp

Kauro: Most doctor’s offices and hospitals have a “Medical Records” office or a patient portal where notes and test results can be sent directly to the patient.

Kauro: Our study staff can help you take the next steps, if necessary

---

Node ID: AJ5y8rf

Parents: nUA9owA

Children: i83McKa

User Transcript: I see

Node ID: 6wCXYUp

Parents: LUuSmzi, nUA9owA

Children: ikGZqyF

User Transcript: Where do I get my genetic testing data?

Node ID: ikGZqyF

Parents: 6wCXYUp

Children: eX5Kmqt, SvnYgFC

Kauro: Usually, you will provide a signed medical release to us, which we will send to the lab that did the original testing or to your doctor’s office. Our study team can help you take the next steps, if necessary

---

Node ID: eX5Kmqt

Parents: ikGZqyF

Children: i83McKa

User Transcript: Okay

Node ID: SvnYgFC

Parents: ikGZqyF

Children: nUA9owA

User Transcript: Where do I get my doctor’s notes?

Node ID: HQwx7B3

Parents: M2fEy3b

Children: 2SpZFSY

User Transcript: Who should I talk to?

Node ID: 2SpZFSY

Parents: HQwx7B3

Children: YyMv36M

Kauro: Some people you could talk to about your participation in this study include your family members, your doctor, or someone you trust to help you make decisions

---

Node ID: YyMv36M

Parents: 2SpZFSY

Children: 85NXhpr

User Transcript: Okay

Node ID: UXhKLb9

Parents: ApKCtVL

Children: Gb4EZXT

User Transcript: How many people will be involved?

Node ID: Gb4EZXT

Parents: UXhKLb9

Children: Gs6eCRA

Kauro: We are hoping to enroll up to 4,200 people.

Kauro: We expect half to be patients and family members recruited at UCI and half to be referred from outside of UCI

Node ID: Gs6eCRA

Parents: Gb4EZXT

Children: M2fEy3b

User Transcript: Got it

Node ID: HUAk3zG

Parents: NHPvQRe

Children: Gufd5fp

User Transcript: What if I am a student or employee at UCI?

Node ID: Gufd5fp

Parents: 8BCaaXA, HUAk3zG

Children: aPfw9zP, k9yQNWE

Kauro: If you are an employee or student (undergraduate, graduate, medical) in training at UCI, your decision to participate or not participate will not affect your employment or academic standing

---

Node ID: aPfw9zP

Parents: Gufd5fp

Children: ckDLoqi

User Transcript: Great!

Node ID: k9yQNWE

Parents: Gufd5fp

Children: aJ5R3Ko

User Transcript: How do I let you know if I no longer want to be in the study?

Node ID: aJ5R3Ko

Parents: Ua23Tdf, k9yQNWE

Children: dYrzLWz, 8BCaaXA

Kauro: You can contact the study team at any time by emailing GREGOR@hs.uci.edu

Kauro: To withdraw from the study, you must send a written letter to the Principal Investigator (Dr. Eric Vilain) to inform him of your decision

---

Node ID: dYrzLWz

Parents: aJ5R3Ko

Children: ckDLoqi

User Transcript: Sounds good!

Node ID: 8BCaaXA

Parents: aJ5R3Ko

Children: Gufd5fp

User Transcript: What if I am a student or employee at UCI?

Node ID: Ua23Tdf

Parents: NHPvQRe

Children: aJ5R3Ko

User Transcript: How do I let you know if I no longer want to be in the study?

Node ID: LbgZ4YK

Parents: SgDDtr8

Children: ZjLbVa4

User Transcript: Tell me more about the different types of genetic test results?

Node ID: ZjLbVa4

Parents: LbgZ4YK

Children: h2xWJaa

Kauro: Of course!

Kauro: When genetic testing is done, we are usually looking for a genetic variant or variants to explain the patient's health concerns

Kauro: When the testing finds an answer, meaning a genetic variant(s) that explains your health concerns is identified, we call this a "positive" result

---

Node ID: h2xWJaa

Parents: ZjLbVa4

Children: WAkoAEV

User Transcript: Okay

Node ID: WAkoAEV

Parents: h2xWJaa

Children: DKTqMXc

[redacted]

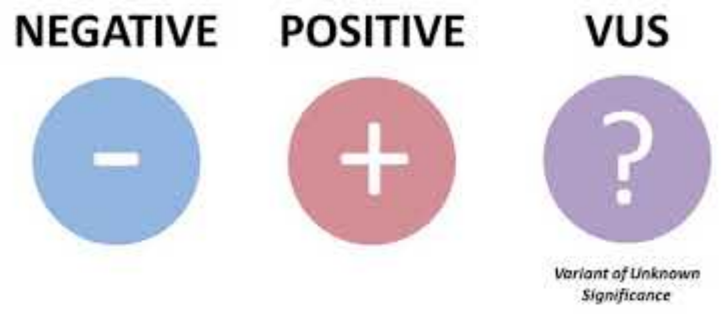

[redacted]

Kauro: Video link: https://www.youtube-nocookie.com/embed/HbQTI2pDmSs

Kauro: If the testing does NOT find any variants that could explain your health concern, we call this a “negative” result

Kauro: Sometimes, the testing can find something that we do not fully understand

Kauro: When a variant is found, but we aren’t sure whether it is linked to a symptom, we call this a “variant of uncertain significance” or VUS

Kauro: Here is a brief video to review the types of genetic results we just discussed:

Node ID: DKTqMXc

Parents: WAkoAEV

Children: XKshQvX

User Transcript: Ah, okay

Node ID: 9cuqxwT

Parents: jczufDn

Children: nVUpS6V

User Transcript: Had genetic testing

Node ID: nVUpS6V

Parents: 9cuqxwT

Children: kYjpLjd

Kauro: So you’ve had some experience. That’s good!

Node ID: kYjpLjd

Parents: nVUpS6V

Children: FiRFHGF

Kauro: One goal for our PMGRC study is to discovery new causes of health problems, like finding a new gene or new ways that differences in our genes can cause health concerns

Kauro: If genetic testing didn't find an answer before, our research might be able to find something new

---

Node ID: iVCEBuL

Parents: EE6kxBa

Children: 8Z6qtgu

User Transcript: That makes sense

Node ID: 9ZmfvBQ

Parents: 45kz8CE

Children: BF3k6W7

User Transcript: Tell me more

Node ID: QXNa2cz

Parents: Ey8gJrc, WnXoffX

Children: abx8PPP

User Transcript: Let's keep it simple

Node ID: abx8PPP

Parents: QXNa2cz

Children: jUSVMUz, W5Gtph5

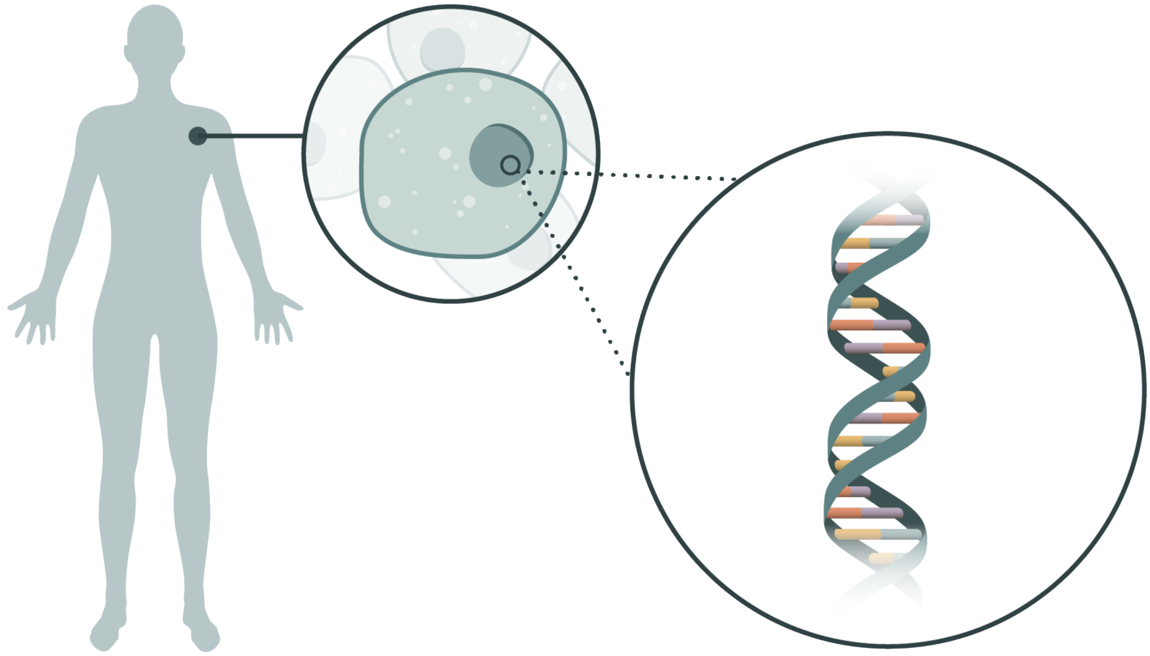

Kauro: Okay, sounds good

Kauro: You may already know that your body is made of trillions of cells. DNA provides the instructions to tell our bodies how to grow and develop

---

Node ID: jUSVMUz

Parents: abx8PPP

Children: 2wRsFE8

User Transcript: Okay

Node ID: 2wRsFE8

Parents: W5Gtph5, jUSVMUz

Children: VNSy2sy

Kauro: Genes are a big part of what makes you who you are

Kauro: In your cells, each gene provides the instructions for molecules called RNA, and each RNA makes a different protein

---

Node ID: VNSy2sy

Parents: 2wRsFE8

Children: 6cFB5vN

User Transcript: That's interesting

Node ID: 6cFB5vN

Parents: VNSy2sy

Children: eo5YNAs, fFHd2uw

Kauro: Your body uses proteins to do many important things

Kauro: For example, some proteins build our tissues, like muscles and nerves. Other proteins help our body break down food and make energy

---

Node ID: eo5YNAs

Parents: 6cFB5vN

Children: jbTfjLx

User Transcript: Okay

Node ID: jbTfjLx

Parents: fFHd2uw, eo5YNAs

Children: TXdMNWZ

Kauro: We all have small differences in our genes, which we call "variants"

Node ID: TXdMNWZ

Parents: jbTfjLx

Children: PiTC5fc

User Transcript: I see

Node ID: PiTC5fc

Parents: TXdMNWZ

Children: Ti3ZavP

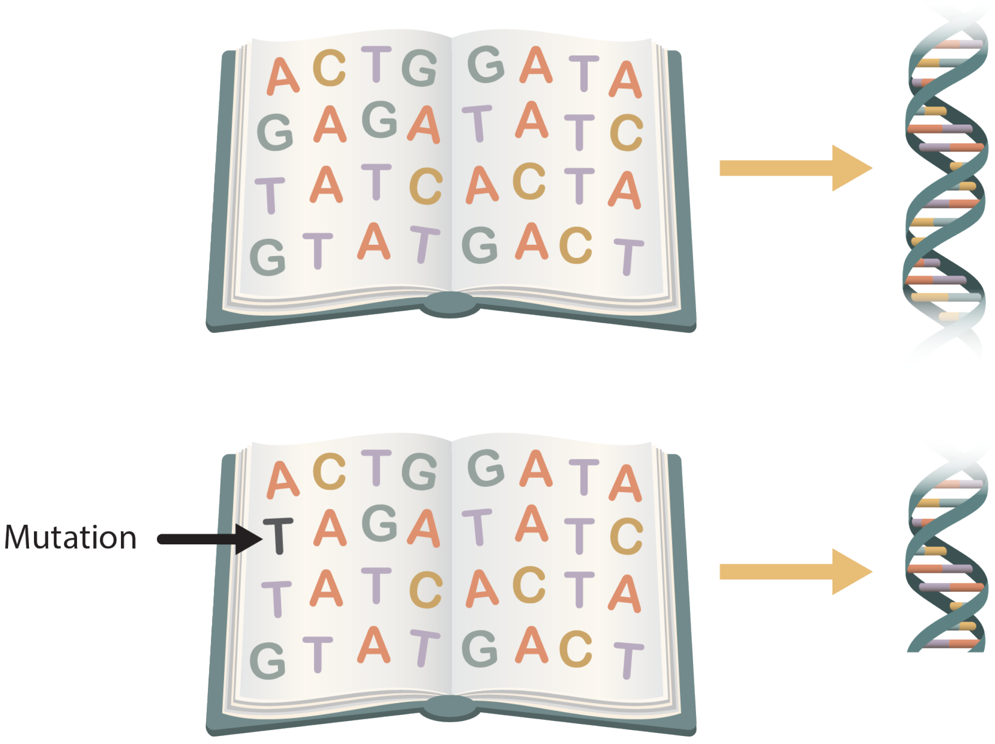

Kauro: When a gene has a variant it gives the body different instructions. It's like changing an ingredient in a recipe

---

Node ID: Ti3ZavP

Parents: PiTC5fc

Children: EE6kxBa

User Transcript: Got it

Node ID: fFHd2uw

Parents: 6cFB5vN

Children: jbTfjLx

User Transcript: That's cool!

Node ID: W5Gtph5

Parents: abx8PPP

Children: 2wRsFE8

User Transcript: This sounds familiar

Node ID: WnXoffX

Parents: AXCfXjh, 4iXP7YP, Sj26JXx

Children: KR89wri, QXNa2cz

Kauro: Now, let's take a moment to talk a little bit about genetics.

Kauro: Do you want to keep it simple or get a little more science-y?

Node ID: VDz6Yx8

Parents: RMb2hrx

Children: 3jPsush

User Transcript: One child

Node ID: 3jPsush

Parents: VDz6Yx8, 5ZrtE33

Children: GkgFmXm

Kauro: Okay, can you please select the ages of your children?

Node ID: Sj26JXx

Parents: DuLoHj9, fmNaDgk, WtmwJYx, mGbcBU9, 5hX84fN

Children: WnXoffX

Kauro: You're welcome 😀

Node ID: 5ZrtE33

Parents: RMb2hrx

Children: 3jPsush

User Transcript: More than one child

Node ID: CAwKwCW

Parents: CPf9CCz

Children: USfk36C

User Transcript: I understand

Node ID: Bkyp7hD

Parents: FADCStr

Children: QT8Mx3R

User Transcript: Why only those related by blood?

Node ID: QT8Mx3R

Parents: Bkyp7hD

Children: RXBDyqd

Kauro: Having genetic data from blood relatives can help us understand genetic findings

Kauro: For example, if we find a genetic change in a person who has specific medical concern, like heart disease, it can be helpful to know if this genetic change was passed down through the family to identify other individuals at risk

---

Node ID: RXBDyqd

Parents: QT8Mx3R

Children: KMirb2i

User Transcript: Ah, that makes sense

Node ID: BxD4wrq

Parents: S2nH9zZ

Children: jbs93Hc

User Transcript: I want to pick up this chat with Kauro sometime later

---

Node ID: jbs93Hc

Parents: BxD4wrq

Children: 87q8gWG

Kauro: Okay! No problem

Kauro: You can use the link in your text message to come back at any time

Kauro: Let me know when you are ready to chat

Node ID: 87q8gWG

Parents: jbs93Hc

Children: Q8yaxQc

User Transcript: I’m ready to chat now

Node ID: iLpXdAS

Parents: oS9m8Ua

Children: a6JKRdh

User Transcript: I understand

Enroll Form

Node ID: b5nYNf6

Parents: USfk36C

Children: eca6cQF, axvGUBt, RMb2hrx

Form ID: checkbox-form

From Text:

From Options:


```
[
  {
    "name": "myself",
    "label": "Myself",
    "value": "Myself",
    "id_value": "eca6cQF"
  },
  {
    "name": "myChildChildren",
    "label": "My child/children",
    "value": "My child/children",
    "id_value": "RMb2hrx"
  },
  {
    "name": "childOtherParent",
    "label": "My child\u2019s other parent",
    "value": "My child\u2019s other parent",
    "id_value": "axvGUBt"
  },
  {
    "name": "adultFamilyMember",
    "label": "Another adult family member",
    "value": "Another adult family member",
    "id_value": "axvGUBt"
  }
]
```

Node ID: eca6cQF

Parents: b5nYNf6

Children: L5xPJtN

Kauro: Great!

Kauro: You can complete the consent process for **yourself** today in this chat

---

Node ID: L5xPJtN

Parents: eca6cQF

Children: Ey8gJrc

User Transcript: Okay, thanks

Node ID: axvGUBt

Parents: b5nYNf6

Children: mBdVTa8, 8dehgq7

Kauro: If your child's other parent or another adult family member is considering enrolling, they should also complete this consent chat themselves

Kauro: If they have not already received a link to this consent chat, I can send them one now with just a few pieces of information

---

Node ID: mBdVTa8

Parents: axvGUBt

Children: AXCfXjh

User Transcript: They have already received a link to this chat

---

Node ID: AXCfXjh

Parents: mBdVTa8

Children: WnXoffX

Kauro: Okay, great

Node ID: 8dehgq7

Parents: axvGUBt

Children: aaVu2Vp

User Transcript: I'd like to send them a link to the chat

Node ID: aaVu2Vp

Parents: 8dehgq7

Children: gWJxSfh

Kauro: Okay!

Kauro: Can you tell me their name and cell phone number and/or email address?

Node ID: gWJxSfh

Parents: aaVu2Vp

Children: UiJSrG3

Form ID: contact-other-adult-form

From Text:

From Options:


```
[
  {
    "name": "firstname",
    "type": "text",
    "label": "First name"
  },
  {
    "name": "lastname",
    "type": "text",
    "label": "Last name"
  },
  {
    "name": "phone",
    "type": "tel",
    "label": "Phone",
    "pattern": "[0-9]{3}-[0-9]{3}-[0-9]{4}",
    "placeholder": "555-867-5309"
  },
  {
    "name": "email",
    "type": "email",
    "label": "Email",
    "placeholder": "example@example.com"
  }
]
```

Node ID: UiJSrG3

Parents: gWJxSfh

Children: 4iXP7YP

Kauro: Thank you! We'll contact them with the information you've provided

Kauro: If you have other adult family members who are considering enrolling, please let our study team know by emailing us at **GREGOR@hs.uci.edu**

Node ID: 4iXP7YP

Parents: UiJSrG3

Children: WnXoffX

User Transcript: Okay, thanks

Node ID: RMb2hrx

Parents: b5nYNf6

Children: VDz6Yx8, 5ZrtE33

Kauro: How many of your children are you considering enrolling?

Test User Understanding

Node ID: VWzWNAK

Parents: bmorWZo, G3EXSPz

Children: c3jUXWd, 6PJ8q9D

Kauro: Question 1: Are you required to participate in the PMGRC study?

Node ID: c3jUXWd

Parents: VWzWNAK

Children: YxYN9RX

User Transcript: No - it’s voluntary

Node ID: YxYN9RX

Parents: c3jUXWd

Children: hfQYPNY

Kauro: That’s right!

Node ID: hfQYPNY

Parents: YxYN9RX, Yd6C4UC

Children: KyvB8Qe, 9LwDBiM

Kauro: Question 2: Is there an alternative to enrolling in the study?

Node ID: KyvB8Qe

Parents: hfQYPNY

Children: 9nbunFh

User Transcript: Yes - the alternative is to not enroll

Node ID: 9nbunFh

Parents: KyvB8Qe

Children: aBLobjJ

Kauro: That’s correct

Node ID: aBLobjJ

Parents: 2N84yDZ, 9nbunFh

Children: BZCE5J4, KmYUkXf

Kauro: Question 3: Is there any cost to you or your insurance provider to participate in the study?

Node ID: BZCE5J4

Parents: aBLobjJ

Children: H6z7NJ9

User Transcript: Yes - I must pay (or have my insurance pay) to participate

---

Node ID: H6z7NJ9

Parents: BZCE5J4

Children: 3fndF8Q

Kauro: Actually, there will be no charge to you or your insurance for this study

Kauro: Participation in this study is free

Node ID: 3fndF8Q

Parents: H6z7NJ9

Children: nWC8AGV

User Transcript: Okay, good to know

Node ID: nWC8AGV

Parents: 3fndF8Q, PBhQ5Mj

Children: EgapBwq, oXEsSu9

Kauro: Question 4: Will you be paid for your participation?

Node ID: EgapBwq

Parents: nWC8AGV

Children: TEL5iQi

User Transcript: No

Node ID: TEL5iQi

Parents: EgapBwq

Children: LFgk3AZ

Kauro: That’s correct

Node ID: LFgk3AZ

Parents: TEL5iQi, BRcszVa

Children: cCwcBvK, DViRS7T, enKSA2K

Kauro: Question 5: Will you benefit from participating in this study?

Node ID: cCwcBvK

Parents: LFgk3AZ

Children: mye4BDq

User Transcript: Yes, definitely

Node ID: mye4BDq

Parents: cCwcBvK

Children: YmLFWDr

Kauro: Actually, that’s not right

Kauro: Though there may be some direct benefits to you, it is also possible that you may not benefit from the study

Kauro: For example, it is possible you may learn about a genetic result that leads to a diagnosis or a better understanding of your health

Kauro: But you may not get any results from this study or any direct benefit

---

Node ID: YmLFWDr

Parents: mye4BDq

Children: hHuaG87

User Transcript: Okay, I understand

Node ID: hHuaG87

Parents: YmLFWDr, fcsbW4h, UpyppKe

Children: mcjM5XL, HSHVE6v

Kauro: Question 6: Are there any risks from participating?

Node ID: mcjM5XL

Parents: hHuaG87

Children: PUv6WAA

User Transcript: Yes, there may be risks

Node ID: PUv6WAA

Parents: mcjM5XL

Children: 2Bh3yqe

Kauro: You're right, there could be some risks

Node ID: 2Bh3yqe

Parents: PUv6WAA, j4CkZ6q

Children: BnpCPkN, Yj34Tnd

Kauro: Question 7: What is the purpose of the study?

Node ID: BnpCPkN

Parents: 2Bh3yqe

Children: FfBKuvU

User Transcript: To get as many samples as possible

Node ID: FfBKuvU

Parents: BnpCPkN

Children: 7zpKYiG

Kauro: That's actually not correct

Kauro: The goal of the study is to discover the causes of genetic health issues and better understand the role of genetic variants

---

Node ID: 7zpKYiG

Parents: FfBKuvU

Children: Wi8Xzvj

User Transcript: Okay, I understand

Node ID: Wi8Xzvj

Parents: 7zpKYiG, 7YuvMHb

Children: b3HAamY, mh5Ta4t

Kauro: Question 8: Will you need to provide samples and/or data?

Node ID: b3HAamY

Parents: Wi8Xzvj

Children: XskwUVW

User Transcript: Yes, the study team may collect some samples and/or data

---

Node ID: XskwUVW

Parents: b3HAamY

Children: JLwjXJk

Kauro: That’s correct

Node ID: JLwjXJk

Parents: XskwUVW, RnQfQa9

Children: TBaxMHp, Mc6Kfcw

Kauro: Question 9: Will your personal identifiable data be kept confidential?

Node ID: TBaxMHp

Parents: JLwjXJk

Children: 22qZY38

User Transcript: Yes, the study team will work to keep my information confidential

---

Node ID: 22qZY38

Parents: TBaxMHp

Children: NPkVfwh

Kauro: That’s right

Node ID: NPkVfwh

Parents: 22qZY38, F276og8

Children: W3wVKub, DiTdvf2

Kauro: Question 10: If you are injured as part of your study participation, what can you do?

Node ID: W3wVKub

Parents: NPkVfwh

Children: ebyungi

User Transcript: Nothing, I have to figure it out myself

Node ID: ebyungi

Parents: W3wVKub

Children: eK9JBuV

Kauro: Actually, that’s not true

Kauro: If you get hurt or sick because of something to do with the study, please call the Principal Investigator, Eric Vilain, at 949-824-4090

Node ID: eK9JBuV

Parents: ebyungi

Children: Qs4fMgg

User Transcript: Okay, now I understand

Node ID: Qs4fMgg

Parents: eK9JBuV, 7ttaVVT

Children: nFpTPVg

Kauro: Thanks for answering these questions!

Kauro: Let me get your results...

Node ID: nFpTPVg

Parents: Qs4fMgg

Children: VUipQHn, G3EXSPz, iH6N9fF

User Transcript: Okay 👍

Node ID: kZ6qj4C

Parents: G3EXSPz

Children: VWzWNAK

User Transcript: Retry the questions.

Node ID: DiTdvf2

Parents: NPkVfwh

Children: 7ttaVVT

User Transcript: I can contact the principal investigator

Node ID: 7ttaVVT

Parents: DiTdvf2

Children: Qs4fMgg

Kauro: Yes, exactly

Node ID: Mc6Kfcw

Parents: JLwjXJk

Children: 97RE52j

User Transcript: No, any of my information may be shared publicly

---

Node ID: 97RE52j

Parents: Mc6Kfcw

Children: F276og8

Kauro: Actually, that's not true

Kauro: The study team will take care to protect the privacy and confidentiality of your study data

---

Node ID: F276og8

Parents: 97RE52j

Children: NPkVfwh

User Transcript: Okay, that's good to know

Node ID: mh5Ta4t

Parents: Wi8Xzvj

Children: UL94XoE

User Transcript: No, no samples or data are ever needed

Node ID: UL94XoE

Parents: mh5Ta4t

Children: RnQfQa9

Kauro: That's not quite true

Kauro: In fact, the study team may need to gather some samples and data about you

Kauro: If you enroll, our study team will be collecting information such as health history, genetic data, and clinical records for anyone who is enrolled in this study

Kauro: You may also be asked to provide a sample for genetic testing, including a blood sample or a cheek swab

---

Node ID: RnQfQa9

Parents: UL94XoE

Children: JLwjXJk

User Transcript: Okay, that makes sense

Node ID: Yj34Tnd

Parents: 2Bh3yqe

Children: 7YuvMHb

User Transcript: To discover the causes of genetic health issues and better understand the role of genetic variants

---

Node ID: 7YuvMHb

Parents: Yj34Tnd

Children: Wi8Xzvj

Kauro: Yes, that's correct

Node ID: HSHVE6v

Parents: hHuaG87

Children: 7YDYb3e

User Transcript: There are no possible risks

Node ID: 7YDYb3e

Parents: HSHVE6v

Children: j4CkZ6q

Kauro: Actually, there are some risks of participating in the study

Kauro: The main risks of this study are related to sample collection, the potential for loss of confidentiality of your information, and possible psychological risks associated with genetic information

---

Node ID: j4CkZ6q

Parents: 7YDYb3e

Children: 2Bh3yqe

User Transcript: I understand

Node ID: DViRS7T

Parents: LFgk3AZ

Children: kYibBDf

User Transcript: No, definitely not

Node ID: kYibBDf

Parents: DViRS7T

Children: UpyppKe

Kauro: Actually, that's not right

Kauro: Though we can't be sure whether you will benefit from participating in the study, it is possible that you will benefit

Kauro: For example, it is possible you may learn about a genetic result that leads to a diagnosis or a better understanding of your health

---

Node ID: UpyppKe

Parents: kYibBDf

Children: hHuaG87

User Transcript: Okay, I understand

Node ID: enKSA2K

Parents: LFgk3AZ

Children: fcsbW4h

User Transcript: Maybe, but not necessarily

Node ID: fcsbW4h

Parents: enKSA2K

Children: hHuaG87

Kauro: Yep, that's right

Kauro: Though we can't be sure whether you will benefit from participating in the study, it is possible that you will benefit

---

Node ID: oXEsSu9

Parents: nWC8AGV

Children: JsvPiaf

User Transcript: Yes

Node ID: JsvPiaf

Parents: oXEsSu9

Children: BRcszVa

Kauro: Actually, that's not correct

Kauro: You will not be paid for your participation in the study

---

Node ID: BRcszVa

Parents: JsvPiaf

Children: LFgk3AZ

User Transcript: Okay, now I see

Node ID: KmYUkXf

Parents: aBLobjJ

Children: PBhQ5Mj

User Transcript: No - participation is free

Node ID: PBhQ5Mj

Parents: KmYUkXf

Children: nWC8AGV

Kauro: That's right!

Node ID: 9LwDBiM

Parents: hfQYPNY

Children: JqyNDaX

User Transcript: No - the only option is to enroll

Node ID: JqyNDaX

Parents: 9LwDBiM

Children: 2N84yDZ

Kauro: Actually, that's not true

Kauro: You have the option to decide NOT to enroll in the study, and this will not impact the medical care you or your family receive

---

Node ID: 2N84yDZ

Parents: JqyNDaX

Children: aBLobjJ

User Transcript: I understand

Node ID: 6PJ8q9D

Parents: VWzWNAK

Children: PWjf47L

User Transcript: Yes - it's required

Node ID: PWjf47L

Parents: 6PJ8q9D

Children: Yd6C4UC

Kauro: Actually, that's not true

Kauro: Participation in the study is completely voluntary

Node ID: Yd6C4UC

Parents: PWjf47L

Children: hfQYPNY

User Transcript: Okay, I understand

Start Consent

Node ID: JTCaWss

Parents: VUipQHn

Children: QKNbLGv, nbkJ3g7

User Transcript: Okay, sounds good

End Consent

Node ID: AimWGCA

Parents: knbtYck

Children: 5n6QHJ7

Form ID: consent-form

From Text:

I am the study participant or I am authorized to act on behalf of the participant.

I have read this consent form or had it read to me.

I have been invited to take part in a research study. I was told why the research is being done and how long my participation in the study is expected to last.

I was told about what will happen in the study and if there are any procedures or drugs that are experimental.

I was told that taking part in this research is voluntary. I also was told that I can decide not to take part or stop being in it at any time without any penalty to me or any change to the quality of care I receive.

I was told about the risks and possible discomforts of taking part in this research study. I was also informed if there are any possible benefits to me if I am in this study.

I have been given the chance to ask questions about the study, and my questions have been answered. If I have questions later, I can ask one of the people listed in this form.

I agree to take part in this research study.

I will receive a signed copy of this Informed Consent/Parental Permission form to keep.

From Options:


```
[
  {
    "name": "fullname",
    "type": "input",
    "label": "Full name",
    "input_type": "text"
  },
  {
    "name": "consent",
    "type": "checkbox",
    "label": "I consent"
  }
]
```

Deactivate Session

Node ID: YQHvnrA

Parents: aXDDbh2

Children:

Kauro: Thank you for completing the consent process with me today!

Kauro: For security reasons, this link will be deactivated within 24 hours

---

Node ID: 7PxxtTh

Parents: nTBdMds

Children:

Kauro: No problem, thank you for completing the consent process with me today!

Kauro: For security reasons, this link will be deactivated within 24 hours

---

Child Consent

Node ID: eDqsGdn

Parents: YkoAEVe

Children: nm8saM4

Kauro: Okay, we will now collect contact information and enrollment preferences for each child, and then we'll ask for your electronic signature

---

Node ID: nm8saM4

Parents: eDqsGdn

Children: 33yRxHn

User Transcript: Okay

Node ID: 33yRxHn

Parents: nm8saM4

Children: XGa7LFM

Kauro: Please provide your child's information below

Node ID: XGa7LFM

Parents: 33yRxHn

Children: afCcd5B

Form ID: child-consent-contact-form

From Text:

From Options:


```
[
  {
    "name": "firstname",
    "type": "input",
    "label": "First name",
    "required": true,
    "input_type": "text"
  },
  {
    "name": "lastname",
    "type": "input",
    "label": "Last name",
    "required": true,
    "input_type": "text"
  },
  {
```

```
    "name": "phone",
    "type": "input",
    "label": "Phone to use for contact regarding this child",
    "pattern": "[0-9]{3}-[0-9]{3}-[0-9]{4}",
    "required": true,
    "input_type": "tel",
    "placeholder": "555-867-5309"
  },
  {
    "name": "email",
    "type": "input",
    "label": "Email to use for contact regarding this child",
    "required": true,
    "input_type": "email",
    "placeholder": "example@example.com"
  },
  {
    "name": "age_group",
    "type": "select",
    "label": "How old is this child?",
    "options": [
      {
        "label": "6 or younger",
        "value": "<=6"
      },
      {
        "label": "7 to 17",
        "value": "7-17"
      },
      {
        "label": "18+ under my guardianship",
        "value": ">=18 guardianship"
      }
    ],
    "required": true
  }
]
```

Node ID: afCcd5B

Parents: XGa7LFM

Children: SFiuhW9

Kauro: We would like to store your child’s samples (such as genetic material including DNA, RNA, or protein, or cells) in a tissue bank for future research as identified below

Kauro: The biobank is maintained by the study team at UCI

Kauro: Please let us know how you would like your child’s samples to be used

---

Node ID: SFiuhW9

Parents: afCcd5B

Children: Ycq8NGN

Form ID: sample-storage-use-form

From Text:

Can we store your child’s samples for purposes of this research study? (Required for participation in the study)

Can we store your child’s samples in UCI’s biobank for purposes of unrelated research?

From Options:


```
[
  {
    "name": "storeSamplesThisStudy",
    "type": "radio",
    "label": "Study Sample Storage",
    "options": [
      {
        "label": "Yes",
        "value": "yes"
      }
    ]
  },
  {
    "name": "storeSamplesOtherStudies",
    "type": "radio",
    "label": "Other Studies Sample Storage",
    "options": [
      {
        "label": "Yes",
        "value": "yes"
      },
      {
        "label": "No",
        "value": "no"
      }
    ]
  }
]
```

Node ID: Ycq8NGN

Parents: SFiuhW9

Children: 6KTYnrc

Kauro: You should know that you may change your mind at a later time and request that your sample be destroyed

Kauro: If you change your mind and want to request that your sample be destroyed, you must send a written request to:

Eric Vilain, MD, PhD
University of California, Irvine (UCI)
Department of Pediatrics
1003 Health Sciences Rd, Suite 308
Irvine, CA 92617

Node ID: 6KTYnrc

Parents: Ycq8NGN

Children: hbBDr5V

User Transcript: That's good to know

Node ID: hbBDr5V

Parents: 6KTYnrc

Children: VaSzySv

Kauro: We would like to store personal health information collected in this study (such as your child's name, date of birth, contact information, etc.) in a database for future research

Kauro: The database is maintained by the study team at UCI

Kauro: Please let us know how you would like your child's PHI to be stored and used

---

Node ID: VaSzySv

Parents: hbBDr5V

Children: VfXY5LK

Form ID: phi-use-form

From Text:

Can we store your PHI in our database for research related to this study? (Required for participation in the study)

Can we use your stored PHI to contact you about future research opportunities that are not related to this study?

From Options:


```
[
  {
    "name": "storePhiThisStudy",
    "type": "radio",
    "label": "PHI Storage for Study",
```

```
    "options": [
      {
        "label": "Yes",
        "value": "yes"
      }
    ]
  },
  {
    "name": "storePhiOtherStudies",
    "type": "radio",
    "label": "PHI Use for Other Studies",
    "options": [
      {
        "label": "Yes",
        "value": "yes"
      },
      {
        "label": "No",
        "value": "no"
      }
    ]
  }
]
```

Node ID: VfXY5LK

Parents: VaSzySv

Children: ZaZ73e4, J5LzZD4

Kauro: You may recall that we discussed the types of genetic research results that may be found in this study

Kauro: You get to decide which types of results you want to learn about

Kauro: Do you remember the different types of results we discussed, like “primary results” and “medically actionable secondary results”?

Node ID: ZaZ73e4

Parents: VfXY5LK

Children: 3Y8xK6c

User Transcript: Yes, I remember

Node ID: 3Y8xK6c

Parents: KByDnk2, ZaZ73e4

Children: nU9xybq

Kauro: Please complete the form below to let us know what types of results you would like to receive for your child

---

Node ID: nU9xybq

Parents: 3Y8xK6c

Children: ggfpZho

Form ID: result-return-form

From Text:

From Options:


```
[
  {
    "name": "rorPrimary",
    "type": "radio",
    "label": "Do you want to learn about primary results that are found?",
    "options": [
      {
        "label": "Yes",
        "value": "yes"
      },
      {
        "label": "No",
        "value": "no"
      }
    ]
  },
  {
    "name": "rorSecondary",
    "type": "radio",
    "label": "Do you also want to learn about medically actionable secondary
results?",
    "options": [
      {
        "label": "Yes",
        "value": "yes"
      },
      {
        "label": "No",
        "value": "no"
      }
    ]
  },
  {
    "name": "rorSecondaryNot",
    "type": "radio",
    "label": "Do you want to learn about secondary results that are NOT
medically actionable?",
    "options": [
```

```
        {
          "label": "Yes",
          "value": "yes"
        },
        {
          "label": "No",
          "value": "no"
        }
      ]
    }
  ]
```

Node ID: ggfpZho

Parents: nU9xybq

Children: nAaeApX

Kauro: Great! You have now entered all of your choices for your child's enrollment

Kauro: You are ready to provide your signature to give consent for your child!

Node ID: nAaeApX

Parents: ggfpZho

Children: Mzke78A, Mzh9KYH

Form ID: consent-form

From Text:

I am the study participant or I am authorized to act on behalf of the participant.

I have read this consent form or had it read to me.

I have been invited to take part in a research study. I was told why the research is being done and how long my participation in the study is expected to last.

I was told about what will happen in the study and if there are any procedures or drugs that are experimental.

I was told that taking part in this research is voluntary. I also was told that I can decide not to take part or stop being in it at any time without any penalty to me or any change to the quality of care I receive.

I was told about the risks and possible discomforts of taking part in this research study. I was also informed if there are any possible benefits to me if I am in this study.

I have been given the chance to ask questions about the study, and my questions have been answered. If I have questions later, I can ask one of the people listed in this form.

I agree to take part in this research study.

I will receive a signed copy of this Informed Consent/Parental Permission form to keep.

From Options:

```
[
  {
    "name": "childname",
    "type": "input",
    "label": "Child\u2019s full name",
    "required": true,
    "input_type": "text"
  },
  {
    "name": "fullname",
    "type": "input",
    "label": "Full name of consenting individual (parent/legal guardian)",
    "required": true,
    "input_type": "text"
  },
  {
    "name": "consent",
    "type": "checkbox",
    "label": "I consent"
  }
]
```

Node ID: J5LzZD4

Parents: VfXY5LK

Children: 9VfACid

User Transcript: No, can you remind me?

Node ID: 9VfACid

Parents: J5LzZD4

Children: eXaerup

Kauro: Primary findings are genetic changes that may be related to the specific health concern that has been seen in your child or your family members. Primary findings often allow the clinical team to make a diagnosis

Kauro: Sometimes the study may find genetic variants that are not related to a patient's current known health concern

Kauro: These are called **secondary findings**. Some secondary findings are considered **medically actionable**, meaning if these genetic changes are known, then you or your healthcare provider can then take action to try to reduce your child's risk of developing symptoms or to better treat or manage the health concern related to the secondary findings

---

Node ID: eXaerup

Parents: 9VfACid

Children: gedo5CN

User Transcript: What kind of findings are medically actionable?

Node ID: gedo5CN

Parents: eXaerup

Children: KByDnk2

Kauro: Some examples of medically actionable secondary findings may be genetic variants that cause increased risk for future health concerns like heart disease or cancer

Kauro: Secondary findings are often related to adult-onset health risks. However, it is possible that we could discover a secondary finding that relates to symptoms in childhood

---

Node ID: KByDnk2

Parents: gedo5CN

Children: 3Y8xK6c

User Transcript: Okay

Follow Up

Node ID: DdxFUae

Parents: S2nH9zZ

Children: nqDwZrd

User Transcript: I'd prefer to talk to someone from the research team

---

Node ID: X4CvZHZ

Parents: iH6N9fF

Children: PZYk7Ga

User Transcript: Okay

Node ID: NsfXTD7

Parents: Dq4hG9k

Children: P8C5vVo

User Transcript: I'm not ready to decide yet

Node ID: BMeYncH

Parents: Dq4hG9k

Children: h9rpY7K

User Transcript: I have more questions before I decide

Decline Consent

Node ID: FVdUisi

Parents: Dq4hG9k, h9rpY7K, ixSPowJ

Children: Swxbt6c

User Transcript: I do not want to consent

Child Age Form

Node ID: GkgFmXm

Parents: 3jPsush

Children: mUpoZUX, gHm22vz, nGiGpjt

Node ID: mUpoZUX

Parents: GkgFmXm

Children: fmNaDgk

Kauro: For your child who is under age 7 years, you will have the option to complete their consent and enrollment during our chat today.

Node ID: fmNaDgk

Parents: mUpoZUX

Children: Sj26JXx

User Transcript: Great, thanks!

Node ID: gHm22vz

Parents: GkgFmXm

Children: gUoc5B6

Kauro: For your child who is between age 7 and age 17, you will have the option to give your consent for your child to enroll in the study

Kauro: However, because your child is over age 6, we may also need to talk to them briefly to tell them about the study. This is called assent

Kauro: If your child is developmentally not able to communicate, you can let us know when you talk with the team

---

Node ID: gUoc5B6

Parents: gHm22vz

Children: eWd55kR

User Transcript: Okay

Node ID: eWd55kR

Parents: gUoc5B6

Children: 5hX84fN

Kauro: So, after you complete the consent chat with me, I can help arrange a time for you (and your child) to talk to a member of our study team to finalize your child's enrollment.

Node ID: 5hX84fN

Parents: eWd55kR

Children: Sj26JXx

User Transcript: That sounds good, thanks

Node ID: nGiGpjt

Parents: GkgFmXm

Children: FMC5dJn, PEgQPMn

Kauro: Is **your 18+ child** still under your legal guardianship?

Node ID: FMC5dJn

Parents: nGiGpjt

Children: AoYLgFK

User Transcript: Yes

Node ID: AoYLgFK

Parents: FMC5dJn

Children: KgLJBEb

Kauro: Okay, after our chat today, you will have the option to give your consent for your child to enroll in the study.

Kauro: But because your child is over age 18, we may also need to speak with them briefly to tell them about the study.

Node ID: KgLJBEb

Parents: AoYLgFK

Children: k5zGiMq

User Transcript: Okay

Node ID: k5zGiMq

Parents: KgLJBEb

Children: DuLoHj9

Kauro: So, after you complete the consent chat with me, I can help arrange a time for you (and your child) to talk to a member of our study team to finalize your child's participation.

Kauro: If your child is developmentally not able to communicate, you can let us know when you talk with our study team.

Node ID: DuLoHj9

Parents: k5zGiMq

Children: Sj26JXx

User Transcript: Okay, thanks

Node ID: PEgQPMn

Parents: nGiGpjt

Children: dFVXKxp

User Transcript: No

Node ID: dFVXKxp

Parents: PEgQPMn

Children: eF87SCm, 3ENuxt7

Kauro: Individuals who are 18 years or older need to complete this consent chat themselves.

Kauro: Does your 18+ child already have a unique link to this chat?

Node ID: eF87SCm

Parents: dFVXKxp

Children: nj5w3by

User Transcript: Yes

Node ID: nj5w3by

Parents: eF87SCm

Children: WtmwJYx

Kauro: Okay, then they will be able to use that link to complete the consent chat themselves.

Node ID: WtmwJYx

Parents: nj5w3by

Children: Sj26JXx

User Transcript: Okay, thanks for letting me know

Node ID: 3ENuxt7

Parents: dFVXKxp

Children: 2b6U7Vy

User Transcript: No, can we send them a link?

Node ID: 2b6U7Vy

Parents: 3ENuxt7

Children: 8A8kAJS, ZykT2RX

Kauro: Sure!

Kauro: Can you tell me your child’s name, cell phone number, and/or email?

Node ID: 8A8kAJS

Parents: 2b6U7Vy

Children: ZykT2RX

Form ID: contact-other-adult-form

From Text:

From Options:


```
[
  {
    "name": "firstname",
    "type": "input",
    "label": "First name",
    "input_type": "text"
  },
  {
    "name": "lastname",
    "type": "input",
    "label": "Last name",
    "input_type": "text"
  },
  {
    "name": "phone",
    "type": "input",
    "label": "Phone",
    "pattern": "[0-9]{3}-[0-9]{3}-[0-9]{4}",
    "input_type": "tel",
    "placeholder": "555-867-5309"
  },
  {
    "name": "email",
    "type": "input",
    "label": "Email",
    "input_type": "email",
    "placeholder": "example@example.com"
  }
```

]

Node ID: ZykT2RX

Parents: 2b6U7Vy, 8A8kAJS

Children: mGbcBU9

Kauro: Thank you! We’ll contact them with the information you’ve provided.

Kauro: If you have other adult family members who are considering enrolling, please let our study team know by emailing us at **GREGOR@hs.uci.edu**

Node ID: mGbcBU9

Parents: ZykT2RX

Children: Sj26JXx

User Transcript: Great, thanks!
